# Heterochromatin and RNAi regulate centromeres by protecting CENP-A from ubiquitin-mediated degradation

**DOI:** 10.1371/journal.pgen.1007572

**Published:** 2018-08-08

**Authors:** Jinpu Yang, Siyu Sun, Shu Zhang, Marlyn Gonzalez, Qianhua Dong, Zhongxuan Chi, Yu-hang Chen, Fei Li

**Affiliations:** 1 Department of Biology, New York University, New York, NY, United States America; 2 State Key Laboratory of Molecular Developmental Biology, CAS Center for Excellence in Biomacromolecules, Institute of Genetics and Developmental Biology, Chinese Academy of Sciences, Beijing, China; Fred Hutchinson Cancer Research Center, UNITED STATES

## Abstract

Centromere is a specialized chromatin domain that plays a vital role in chromosome segregation. In most eukaryotes, centromere is surrounded by the epigenetically distinct heterochromatin domain. Heterochromatin has been shown to contribute to centromere function, but the precise role of heterochromatin in centromere specification remains elusive. Centromeres in most eukaryotes, including fission yeast (*Schizosaccharomyces pombe*), are defined epigenetically by the histone H3 (H3) variant CENP-A. In contrast, the budding yeast *Saccharomyces cerevisiae* has genetically-defined point centromeres. The transition between regional centromeres and point centromeres is considered as one of the most dramatic evolutionary events in centromere evolution. Here we demonstrated that Cse4, the budding yeast CENP-A homolog, can localize to centromeres in fission yeast and partially substitute fission yeast CENP-A^Cnp1^. But overexpression of Cse4 results in its localization to heterochromatic regions. Cse4 is subject to efficient ubiquitin-dependent degradation in *S*. *pombe*, and its N-terminal domain dictates its centromere distribution via ubiquitination. Notably, without heterochromatin and RNA interference (RNAi), Cse4 fails to associate with centromeres. We showed that RNAi-dependent heterochromatin mediates centromeric localization of Cse4 by protecting Cse4 from ubiquitin-dependent degradation. Heterochromatin also contributes to the association of native CENP-A^Cnp1^ with centromeres via the same mechanism. These findings suggest that protection of CENP-A from degradation by heterochromatin is a general mechanism used for centromere assembly, and also provide novel insights into centromere evolution.

## Author summary

Centromere is a specialized chromosomal domain that is essential for faithful chromosome segregation during mitosis and meiosis. In most eukaryotes, centromeres are surrounded by the densely packed heterochromatin. The exact role of heterochromatin in centromere assembly remains elusive. Centromeres in most eukaryotes are defined epigenetically by a conserved histone 3 variant, CENP-A. Ubiquitin-mediated proteolysis prevents mislocalization of CENP-A at non-centromeric regions. In this work, we demonstrated that heterochromatin and RNAi mediate centromere specification by protecting CENP-A from ubiquitin-mediated degradation. This mechanism may provide answers to long-lasting questions, such as why centromeres are usually flanked by heterochromatin, and why neocentromeres often form at heterochromatic regions. Our study also has important implications in centromere evolution. One fascinating aspect of centromere evolution is the transition between epigenetically defined “regional centromeres” and genetically defined “point centromeres”. Our findings suggest that lacking pericentromeric heterochromatin in point centromeres may contribute to the dramatic transition between regional and point centromeres in centromere evolution.

## Introduction

Chromatin is organized into different chromatin domains. Centromere is a specialized chromatin domain that plays a vital role in chromosome segregation [1–3]. In most eukaryotes, centromere is surrounded by the epigenetically distinct heterochromatin domains. Peri-centromeric heterochromatin is usually composed of tandem DNA repeats that are organized into condensed, transcriptionally silenced structures. The region contains the conserved hallmark of heterochromatin, histone H3 lysine 9 (H3K9) methylation [1]. Heterochromatin has been shown to contribute to centromere function [4–9]. But its exact role in centromere assembly remains unknown.

The vast majority of eukaryotes have “regional centromeres”, which contain large regions of DNA, hosting multiple microtubule nucleation sites. DNA sequence in regional centromeres is highly variable among species. Sequence-independent epigenetic mechanisms are crucial for specification of “regional” centromeres. A conserved histone 3 (H3) variant protein, CENP-A, serves as the epigenetic mark for centromeres [1–3]. The histone variant partially replaces the canonical histone H3 in centromeres, and is assembled into unique CENP-A nucleosomes that promote kinetochore assembly. CENP-A proteins across species share a conserved C-terminal histone fold domain containing the CENP-A targeting domain (CATD), while the N terminus tails are vastly divergent in sequence and length [10–12]. It has been proposed that CENP-A evolves adaptively in concert with the centromeric sequence [8, 13].

Unlike other eukaryotes, the budding yeast *Saccharomyces cerevisiae* contain genetically defined “point centromeres”. Point centromeres are only ~125 base pairs in length, and this short DNA sequence is necessary and sufficient for centromere formation. The centromeric DNA is recognized by specific DNA-binding proteins that recruit the CENP-A homolog, Cse4, to drive kinetochore assembly [1, 14, 15]. Noticeably, point centromeres lack peri-centromeric heterochromatin. The transition between regional centromeres and point centromeres is considered as one of the most dramatic evolutionary events in centromere evolution [8]. It has been shown that major heterochromatin proteins and RNAi machinery were lost in budding yeast [16, 17]. The evolution event appears coupled with the emergence of point centromeres. This raised an important question of how epigenetically-defined regional centromere that requires heterochromatin machinery evolved to a genetically defined point centromere that relinquishes the requirement for these proteins [8].

Mistargeting of CENP-A to non-centromeric regions has been reported to produce ectopic kinetochores, and trigger chromosome instability and aneuploidy in multiple organisms. Overexpression of CID, the *Drosophila* CENP-A, promotes formation of ectopic centromere, leading to chromosome missegregation and growth defects [18]. Interestingly, ectopic centromeres prefer to assemble at regions near heterochromatin [19]. In budding yeast *S*. *cerevisiae*, mislocalization of Cse4 also results in defects in chromosome segregation [20]. Overexpression and mispositioning of CENP-A have been found in many cancer cells and contribute to carcinogenesis [21–25]. One of the conserved mechanisms used to prevent CENP-A mispositioning is ubiquitin-dependent proteolysis. Proteolysis of CENP-A in both budding yeast and *Drosophila* ensures the exclusive restriction of CENP-A at centromeres [26–28]. Both the N-terminus and the CATD domain in the conserved C-terminus in the budding yeast Cse4 are important for proteolysis of the protein [29, 30]. A recent study showed that the FACT (facilitates chromatin transcription/transactions) complex mediates the degradation of Cse4 by destabilizing the ectopic Cse4 nucleosomes and facilitating the interaction of the E3 ubiquitin ligase, Psh1, with Cse4 [31]. On the other hand, the kinetochore has been reported to protect Cse4 at point centromeres from ubiquitin-mediated proteolysis [26, 32]. The association of Scm3/HJURP, a CENP-A specific chaperone, was also suggested to play a role in guarding Cse4 at centromeres [27]. In addition, the kinetochore is implicated in protecting of CENP-A degradation in regional centromeres in *Candida albicans* [33]. However, how CENP-A is protected from degradation especially at regional centromeres remains poorly understood.

In contrast to budding yeast, evolutionarily divergent fission yeast (*Schizosaccharomyces pombe*) contains regional centromeres defined by the CENP-A homolog, Cnp1 [1, 34]. Centromeres in fission yeast are also flanked by heterochromatin. Overexpression of Cnp1 can cause chromosome missegregation during both mitosis and meiosis [35–37]. We have found that the N terminus of Cnp1 is important for its ubiquitin-mediated degradation [37]. In addition, centromeres and peri-centromeric heterochromatin are localized near the nuclear membrane periphery, where proteasome subunits, such as Rpt3 and Mts2, and a proteasome anchor protein Cut8 are enriched [38–40]. The pericentromeric heterochromatin in fission yeast is marked by H3K9 methylation. Clr4, a homolog of the mammalian histone methyltransferase SUV39H1, mediates H3K9 methylation, which is bound by the HP1 homolog Swi6. RNA interference (RNAi) plays an important role in H3K9 methylation and heterochromatin silencing [1, 41, 42]. It has been reported that RNAi-mediated heterochromatin is required for Cnp1 assembly at neocentromeres but is dispensable for inheritance of Cnp1 chromatin [6]. Deletion of centromere sequences in fission yeast can result in formation of ectopic centromere in heterochromatin region [5]. We and others have also shown that overexpressed Cnp1 forms ectopic loci that are often associated with heterochromatic regions [35, 37]. But the mechanism underlying the role of heterochromatin in centromere assembly remains unknown.

To study the role of peri-centromeric heterochromatin in centromere specification, we expressed the budding yeast Cse4 in *S*. *pombe*. We demonstrated that Cse4 can target to fission yeast centromeres, but overexpression of Cse4 leads to its preferential localization in heterochromatin. We showed that Cse4 in *S*. *pombe* is subject to efficient ubiquitination and degradation, resulting in its expression at low level. Using Cse4 domain-deletion mutants and also domain-swapped chimeras by swapping the N- and C-terminal domains of Cse4 and Cnp1, we showed that the N-terminal domain of these proteins mediates their proper centromere distribution by dictating the protein level via ubiquitination. Importantly, we found that heterochromatin and RNAi promote targeting of Cse4 to centromeres by protecting Cse4 from ubiquitin-dependent degradation. Peri-centromeric heterochromatin also protects native Cnp1 at centromeres. In heterochromatin mutants, proteasomes are accumulated in centromeres, resulting in high ubiquitination of Cnp1 and its unstable association with centromeres. To our knowledge, our findings provide the first mechanistic insight into the role of heterochromatin in the regulation of centromere specification. The study advances our understanding of how different chromatin domains functionally interact, and also sheds light on centromere evolution.

## Results

### Cse4 can target to centromeres and partially substitute Cnp1 in fission yeast

**Fig 1:**
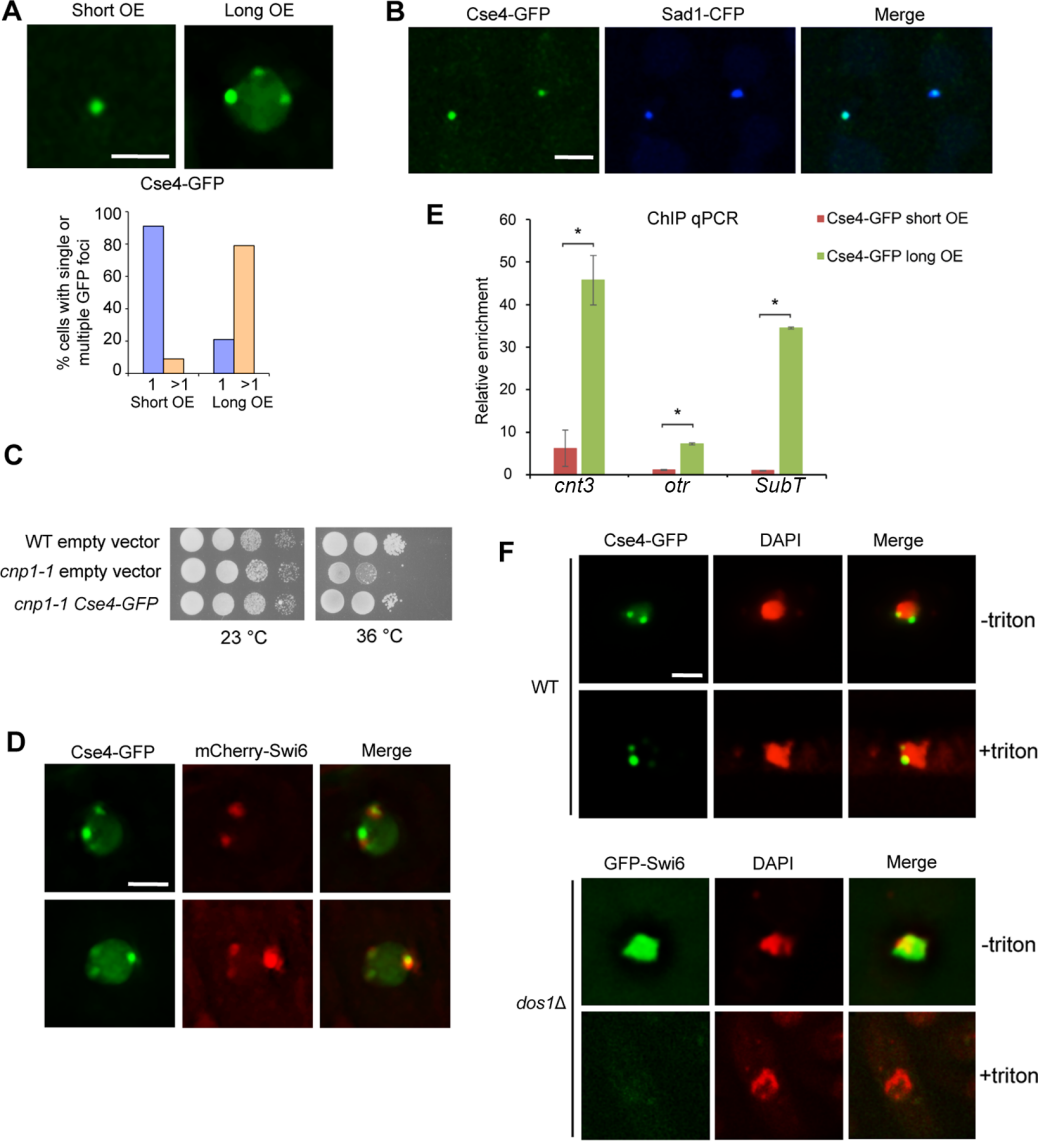
Cse4 preferentially targets to centromeres and functionally substitutes Cnp1 in fission yeast. **A,** Cells carrying pREP1-CSE4-GFP that were induced for 24 hours show a single GFP focus (short). Multiple foci (commonly 3–6) were detected for 28-hour induction (long). (Bottom) Percentage of cells containing single or multiple focus. OE, overexpression. **B,** The single Cse4-GFP focus colocalizes with Sad1-CFP. **C,** Cse4-GFP partially rescues *cnp1-1* growth defects at 36°C. Serial dilutions of indicated strains were plated in minimal medium without thiamine. Dilution = 10. **D,** Multiple GFP foci in cells overexpressing Cse4-GFP colocalize with mCherry-Swi6. **E**. ChIP qPCR showing relative enrichment of Cse4-GFP to centromeric region (*cnt3*), peri-centromeric region (*otr*) and sub-telomeric region (*subT*). ChIP was repeated in triplicate. Error bar indicates SEM. *, p<0.01. **F,** *in situ* chromatin-binding assay for wild type cells expressing Cse4-GFP. The heterochromatin mutant *dos1*Δ carrying GFP-Swi6 was used as a control (Bottom). GFP-Swi6 is dissociated from heterochromatin in *dos1*Δ, and was readily washed away by Triton X-100 as expected. Scale bars: 2 μm.

To investigate how Cse4 behaves in fission yeast, Cse4 was constructed under the strong inducible promoter *nmt1* in the fission yeast expression vector, pREP1. The promoter was induced when thiamine is depleted from the media. We found that no florescent signal in wild-type (WT) cells carrying Cse4-GFP was detected in repressed condition. However, ~90% of cells exhibited a single GFP focus 24 hours after thiamine withdrawal (Fig 1A, left panel). In *S*. *pombe*, centromeres are clustered near the spindle pole body (SPB) during interphase. We found that the single Cse4-GFP focus colocalizes with the CFP-tagged SPB protein Sad1 (Fig 1B), indicating that Cse4-GFP is associated with centromeres. Chromatin immunoprecipitation (ChIP) analysis also confirmed that Cse4-GFP is enriched in the centromeric sequences (Fig 1E). To test whether Cse4 can functionally substitute Cnp1 in fission yeast, Cse4 was expressed in the temperature sensitive (*ts*) mutant, *cnp1-1*. When cultured at 36°C, *cnp1-1* cells are non-viable, but the presence of Cse4 largely complements the growth defects, demonstrating that Cse4 is at least partially substitute Cnp1 to establish functional centromeres in fission yeast (Fig 1C).

### Overexpressed Cse4-GFP is preferentially localized at heterochromatin

We noticed that with extended overexpression, Cse4-GFP was found further enriched in centromeric regions (Fig 1E), which is similar to overexpressed Cnp-GFP (S1 Fig). In addition, we found more Cse4-GFP foci with extended overexpression. 79% of cells at 28-hour induction display multiple foci, commonly 2–6, that tend to be associated with the nuclear envelope (Fig 1A, right panel). Heterochromatin in fission yeast interphase cells is organized into 2–6 clusters next to the nuclear envelope; cells expressing mCherry-tagged Swi6 thus also exhibit 2–6 foci [37, 43]. We found that these Cse4-GFP foci largely colocalize with mCherry-Swi6 (Fig 1D), suggesting that overexpressed Cse4-GFP preferentially binds to heterochromatic regions. This is reminiscent of Cnp1’s enrichment to heterochromatic domains when overexpressed [35, 37]. We also performed ChIP qPCR analysis to quantitate the relative enrichment of Cse4-GFP to heterochromatin regions. Consistent with the microscopic observations, Cse4-GFP is absent in pericentromeric and sub-telomeric heterochromatin regions with short overexpression (Fig 1E). But when the induction of Cse4-GFP is extended for additional 4 hours, Cse4-GFP is enriched in these heterochromatic regions (Fig 1E). To further determine whether Cse4-GFP foci are formed by stable chromatin incorporation of Cse4, we performed *in situ* chromatin-binding assays. GFP-Swi6 in the heterochromatin mutant *dos1Δ* (also known as *raf1Δ*) [44–46] appears diffuse in the nucleus due to the dissociation of Swi6 from chromatin, and the GFP signal can be readily washed away by Triton X-100. But we found that Cse4-GFP is resistant to Triton X-100 extraction (Fig 1F), suggesting that Cse4-GFP is stably associated with chromatin.

### Cse4 undergoes efficient ubiquitination and degradation in fission yeast

**Fig 2:**
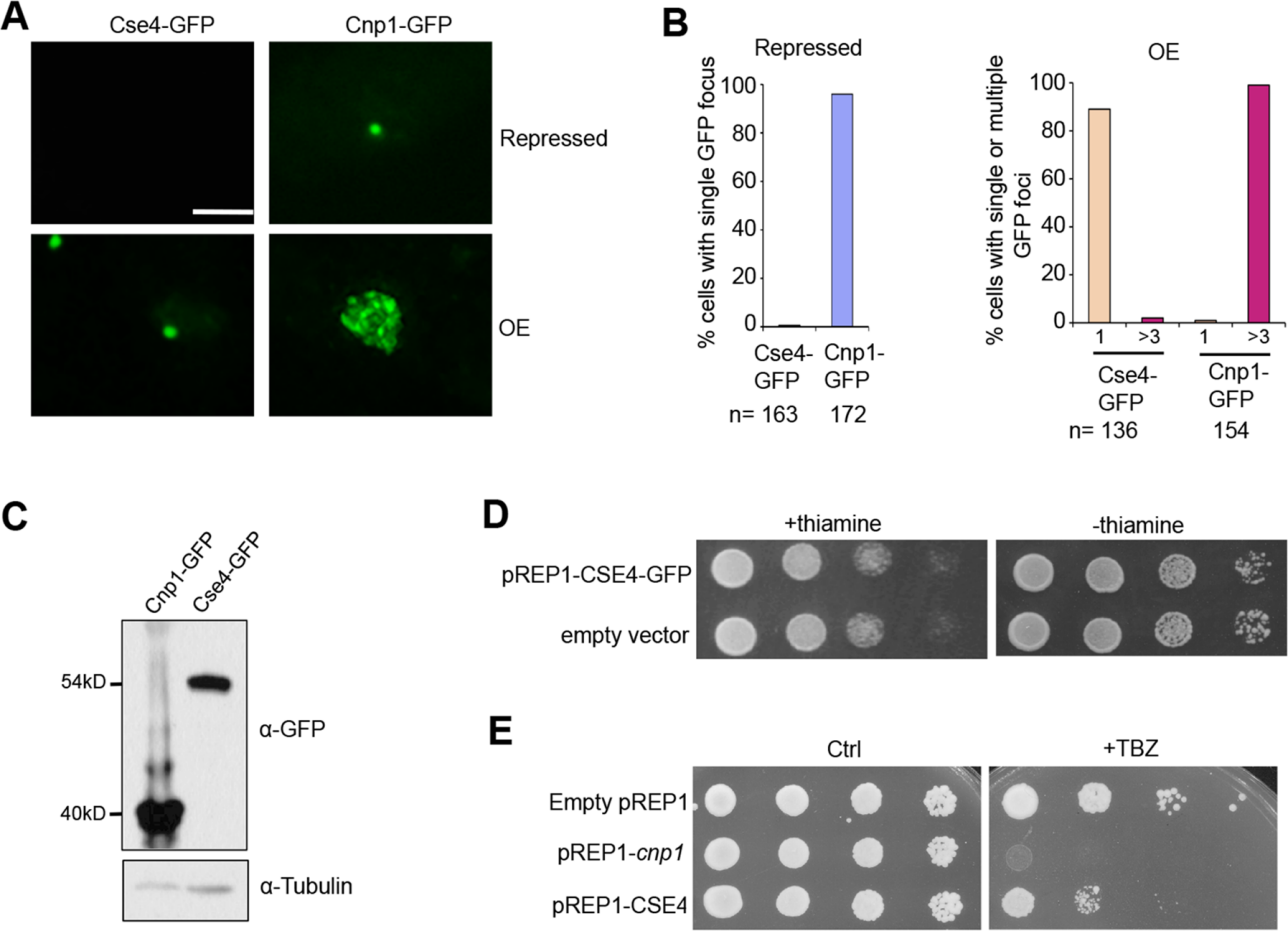
Cse4 is expressed at a significantly low level in the fission yeast. **A,** Distribution patterns of Cse4-GFP and Cnp1-GFP at repressed (Top) and overexpressed (Bottom) conditions (24-hour induction). **B,** Quantification of the percentage of cells showing a single focus when repressed (Left), and a single or multiple GFP foci (>3) in overexpressed condition (Right). **C,** Western blot analysis of cells expressing indicated proteins using an anti-GFP antibody. Tubulin was used as a loading control. **D,** Cells harboring pREP1-CSE4-GFP were grown in minimal medium with thiamine to suppress its expression (Left) or lacking thiamine to induce overexpression (Right). Wild type cells carrying an empty vector were used as a control. **E,** Overexpression of Cse4 results in minor chromosome segregation defects. Cells carrying indicated plasmids were plated in minimal medium that contains 15μg/ml TBZ but no thiamine. Dilution = 10. Ctrl, a control plate without TBZ. Scale bar: 2 μm.

We have shown previously that at the repressed condition, a single GFP focus formed in almost all cells carrying pREP1-Cnp1-GFP due to leaky expression from the promoter [37]. In contrast, no GFP signal was observed in cells carrying pREP1-CSE4-GFP at the same condition (Fig 2A and 2B). In addition, at 24-hour induction, ~90% of cells carrying Cse4-GFP contain a single focus, whereas nearly all the cells overexpressing Cnp1-GFP exhibit multiple bright foci (commonly 6–30), or widespread fluorescent signal (Fig 2A and 2B). Moreover, it takes ~20% more induction time to have Cse4-GFP signal sufficiently induced compared to Cnp1-GFP (S2A Fig), and the additional induction time does not increase their mRNA expression (S2B Fig), suggesting at the same time point Cse4-GFP protein level is lower than Cnp1-GFP. These observations prompted us to examine the level of Cse4 and Cnp1 in these conditions by western blot assays, and we found that protein level of Cse4 is significantly lower than Cnp1 (Fig 2C).

Overexpression of Cnp1 in fission yeast causes chromosome mis-segregation and growth defects [37], while overexpression of Cse4 in budding yeast cells displays little phenotype [26, 47]. We next examined the effect of overexpression of Cse4 in fission yeast. We found that overexpressing Cse4 in fission yeast leads to no obvious growth defects (Fig 2D). Consistently, cells overexpressing Cnp1 is highly sensitive to microtubule-destabilizing drug, thiabendazole (TBZ), but the drug only has minor effect on growth of cells overexpressing Cse4 (Fig 2E). The significantly reduced expression level of Cse4 may, at least partially, explain why overexpressing Cse4 does not exert growth defects as Cnp1 overexpression.

**Fig 3:**
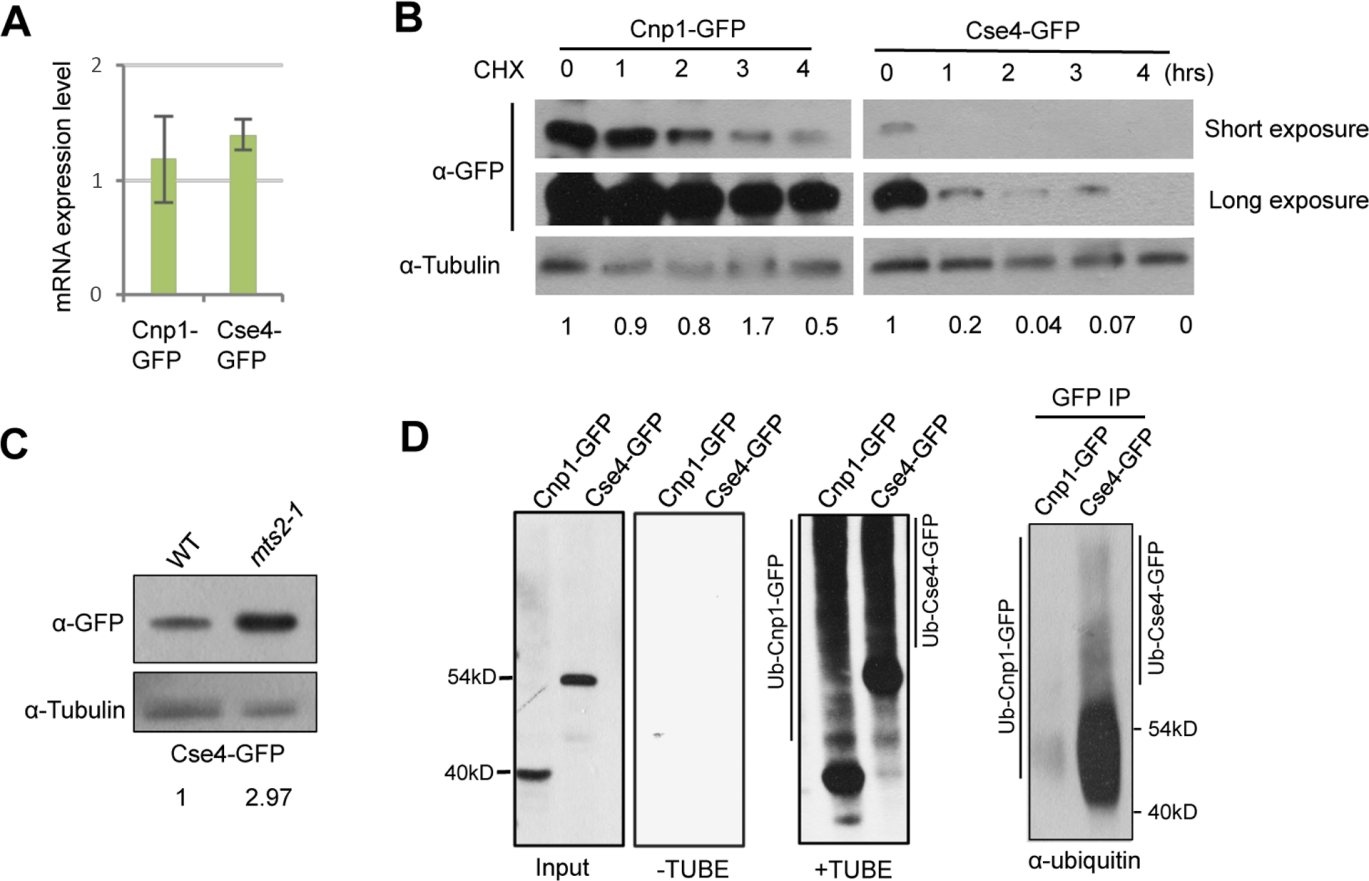
Cse4 is subject to efficient ubiquitin-dependent degradation in the fission yeast. **A,** RT-PCR analysis of cells expressing Cnp1-GFP or Cse4-GFP. Total RNA extracted from cells overexpressing Cnp1-GFP or Cse4-GFP was used. Cnp1-GFP or Cse4-GFP transcripts were analyzed with primers specific for GFP. Actin was used as an internal control. **B,** Lysates from cells collected at indicated time points (hrs) following cycloheximide treatment were analyzed by western blotting with an anti-GFP antibody. **C,** Cse4 level is enhanced after proteasome inactivation in fission yeast. Cells overexpressing Cse4-GFP in wild type or *mts2-1* background were incubated at 37°C for 4 hours, and were subject to western blot analysis using an anti-GFP antibody. Tubulin was used as a loading control. **D,** Extracts from cells expressing indicated proteins were split, and subject to TUBE pull-down and reverse pull-down assays, respectively. For TUBE pull-down assays, extracts were immunoprecipitated with tandem ubiquitin-binding entities (+TUBE), or control Argarose beads (-TUBE), followed by western blot analysis using an anti-GFP antibody. For reverse pull-down assays (right panel), extracts were immunoprecipitated with an anti-GFP antibody, then analyzed by western blotting using a pan ubiquitin antibody. Induction time: 20 hours for Cnp1-GFP; 24 hours for Cse4-GFP.

Both low level of transcription and high rate of protein degradation could contribute to the observed low expression level of Cse4. We first analyzed the transcription level of Cse4-GFP by RT-qPCR, and found it similar to the level of Cnp1-GFP expressed at the same condition (Fig 3A). We next investigated the protein stability of Cse4 and Cnp1 by western blotting. We found that while Cnp1 level persists up to 4 hours after treatment with cycloheximide, an inhibitor of protein synthesis, Cse4 is quickly degraded (Fig 3B).

Cse4 in budding yeast is regulated by ubiquitin-mediated proteolysis [26, 27]. To examine whether Cse4 in fission yeast is also subjected to the degradation pathway, we expressed Cse4-GFP level in cells carrying a *ts* mutant of a 19S proteasome regulatory subunit, *mts2-1*. After 4 hours of exposure to the restrictive temperature, Cse4-GFP protein level is significantly accumulated, indicating that Cse4 in fission yeast is also regulated by ubiquitin-mediated proteolysis (Figs 3C and S3).

To examine the extent to which Cse4 is ubiquitinated, we performed affinity pull-down assays using TUBEs (Tandem Ubiquitin Binding Entity) in *mts2-1* cells expressing Cnp1-GFP or Cse4-GFP. We observed a laddering pattern for both Cnp1 and Cse4 after immunoprecipitation with TUBEs, whereas the control sample immunoprecipitated with empty agarose beads does not show the laddering pattern, indicating that both proteins are polyubiquitylated (Fig 3D). Furthermore, the laddering pattern in cells expressing Cse4-GFP is considerably enhanced, compared with Cnp1-GFP (Fig 3D). This finding was corroborated by a reverse pull-down assay, where Cnp1-GFP and Cse4-GFP were immunoprecipitated with an antibody against GFP and analyzed by western blot analysis using a pan ubiquitin antibody (Fig 3D). We therefore concluded that Cse4 in fission yeast is subject to efficient ubiquitin-dependent degradation, explaining why expression level of Cse4 is significantly lower than the level of Cnp1 in fission yeast.

### N terminus of Cse4 is important for its stability and centromeric localization

Despite sharing a conserved C terminal domain, the N terminus tails of Cse4 and Cnp1 are remarkably different. Cse4 contains a relatively long N-terminus consisting of 130 amino acids, whereas the N-terminus of Cnp1 has only 20 amino acids (S4 Fig). We hypothesized that the N terminus tails of these proteins may contribute to the difference in stability between Cse4 and Cnp1 in fission yeast. To determine the role of Cse4’s N terminal domain, we created a strain carrying N-terminal deleted Cse4-GFP under the *nmt1* promoter, Cse4-NΔ-GFP. We found that deletion of the N-terminal domain of Cse4 results in largely diffused but brighter GFP signal in the nucleus after the 22-hour induction, whereas most of cells carrying Cse4-GFP contain a single GFP focus at the same time (S5A Fig). This, together with western blot analysis (S5B Fig), demonstrated that N-terminal deleted Cse4-GFP exhibits a higher protein level.

**Fig 4:**
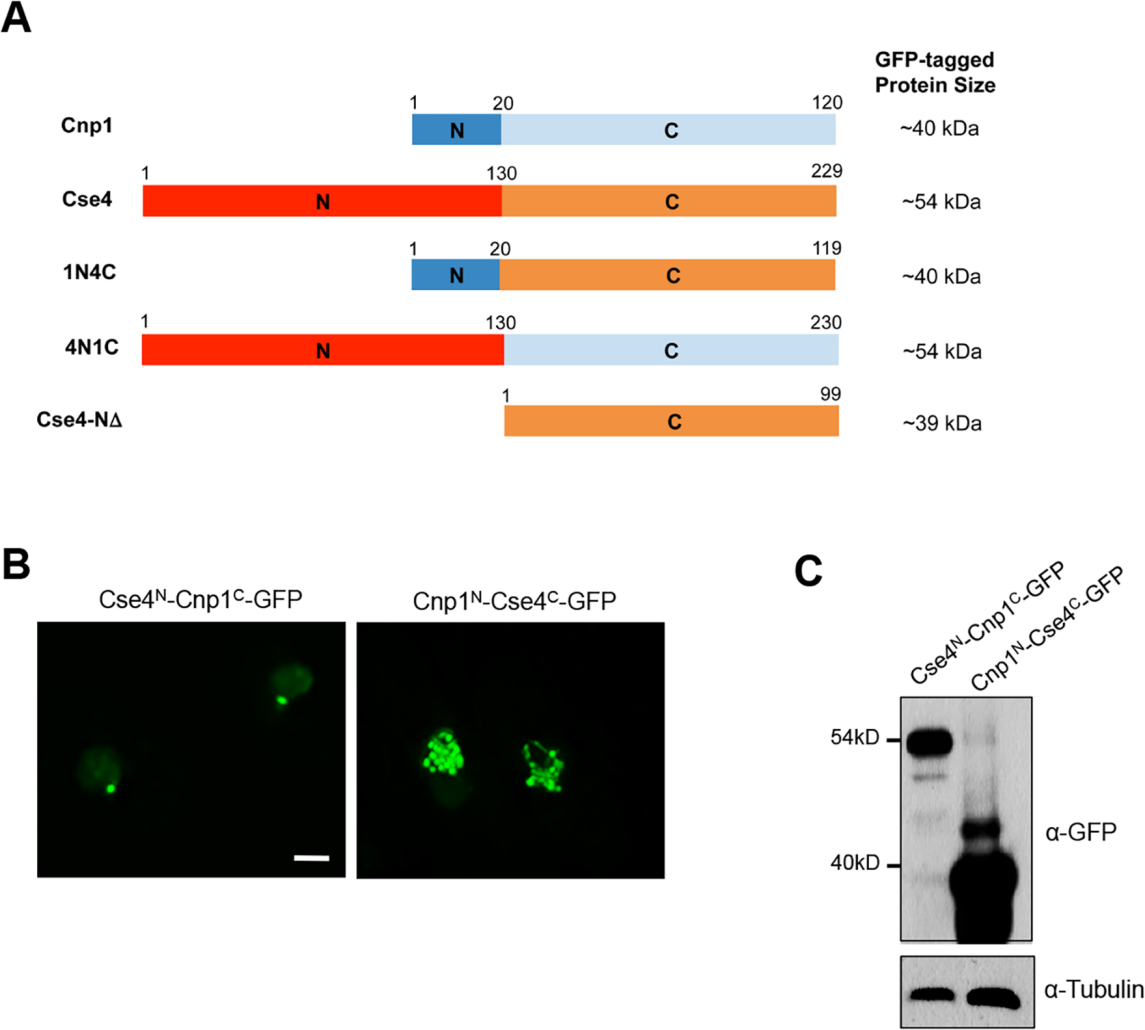
N-terminus of Cse4 is important for its distribution and stability. **A**, Schematic diagram of the domain organizations of Cnp1^N^-Cse4^C^ (1N4C) and Cse4^N^-Cnp1^C^ (4N1C) proteins. Full-length Cnp1, Cse4 and N-terminal deleted Cse4 (Cse4-NΔ) are also shown. **B,** Distribution patterns of overexpressed Cse4^N^-Cnp1^C^-GFP and Cnp1^N^-Cse4^C^-GFP. Induction times: 24 hours. Scale bar: 2 μm. **C,** Western blot analysis of cells expressing indicated proteins using an anti-GFP antibody. Tubulin was used as a loading control.

To further examine the role of Cse4’s N terminus, we fused it with the C-terminal domain of Cnp1 to generate Cse4^N^-Cnp1^C^-GFP. We also constructed a chimera containing the N-terminal domain of Cnp1 and the C-terminus of Cse4, Cnp1^N^-Cse4^C^-GFP (Fig 4A). We found that most cells overexpressing Cse4^N^-Cnp1^C^-GFP contain a single focus 24 hours after induction, similar to cells expressing Cse4-GFP under the same condition. In contrast, expression of Cnp1^N^-Cse4^C^-GFP at the same condition results in multiple foci, or widespread signal throughout the nucleus (Fig 4B), which phenocopied cells overexpressing Cnp1-GFP [37]. Consistent with this, the level of Cse4^N^-Cnp1^C^-GFP is significantly lower than Cnp1^N^-Cse4^C^-GFP (Fig 4C). The N-terminus of Cse4 and Cnp1 thus dictates their protein level and proper distribution.

The N-terminus of both Cse4 and Cnp1 has been implicated in ubiquitin-dependent proteolysis [29, 37]. Accordingly, we found that Cse4-NΔ-GFP expression is more stable by our protein stability assays (S5C Fig). Furthermore, our *in vivo* ubiquitination assays revealed that N-terminus-deleted Cse4 is less ubiquitinated than WT Cse4 (S5D Fig), demonstrating the importance of the domain in ubiquitin-mediated Cse4 degradation in fission yeast. We speculate that the long N terminus of Cse4 may facilitate the recognition by ubiquitin ligases that lead to higher ubiquitination.

### Heterochromatin and RNAi are indispensable for the centromeric association of Cse4

**Fig 5:**
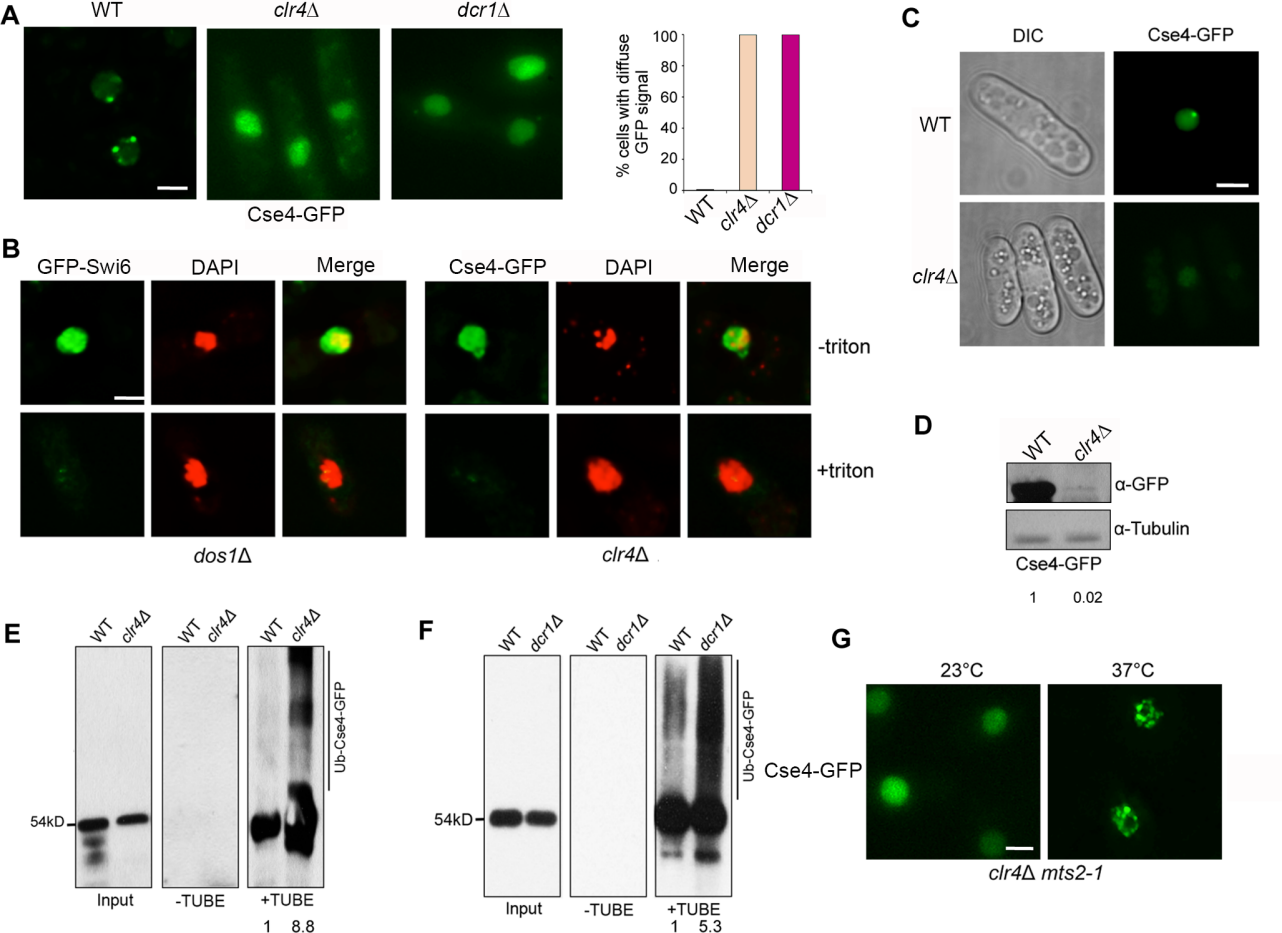
Heterochromatin protects Cse4 at centromeres from ubiquitin-mediated degradation. **A,** Cse4-GFP in *clr4*Δ and *dcr1*Δ cells at 28-hour induction show total diffuse pattern. (Right) Percentage of cells exhibiting a diffuse GFP signal. **B,** *in situ* chromatin-binding assay for *clr4*Δ cells overexpressing Cse4-GFP. (Left) *dos1*Δ mutant carrying GFP-Swi6 was used as a control. **C,** Distribution pattern of Cse4-GFP in WT and *clr4*Δ at 24-hour induction. **D,** Western blot analysis of Cse4-GFP in WT and *clr4*Δ. Induction time: 28 hours. **E, F,** Extracts from indicated cells were analyzed by TUBE assays. Induction time: 24 hours for Cse4-GFP and 28 hours for Cse4-GFP *clr4*Δ. **G,** Distribution pattern of overexpressed Cse4-GFP in the *clr4*Δ *mts2-1* double mutant incubated at 37°C for 4 hours. Cells were induced for 40 hours at 23°C, then switched to permissive or restrictive temperature for additional 4 hours. Scale bars: 2 μm.

While regional centromeres are flanked with heterochromatin, point centromeres in budding yeast lack pericentromeric heterochromatin. To investigate whether the centromeric association of Cse4-GFP is affected by heterochromatin, we examined the distribution of Cse4-GFP in the *clr4*Δ mutant. Remarkably, we found that Cse4-GFP in *clr4*Δ is completely diffused in almost all cells observed (Figs 5A and S6). We also observed the same diffuse Cse4-GFP pattern in the Dicer mutant, *dcr1*Δ (Figs 5A and S6). To determine whether diffused Cse4-GFP in the *clr4*Δ is chromatin-bound, we performed *in situ* chromatin-binding assays. We found that, whereas Cse4-GFP in the wild-type cells forms stable foci that are resistant to Triton X-100 extraction, Cse4-GFP in *clr4*Δ is effectively washed away from the nucleus by the treatment (Fig 5B), indicating that Cse4-GFP in the *clr4*Δ does not assemble stably with chromatin. These data suggest that heterochromatin promotes chromatin assembly and centromeric targeting of Cse4 in fission yeast.

### Heterochromatin protects Cse4 from ubiquitin-mediated degradation

Aside from lacking centromeric targeting, we also observed that the Cse4-GFP signal is very faint in *clr4*Δ cells with Cse4-GFP fully induced for 28 hours, indicating that the protein is expressed at low level. Consistent with this, at 24-hour induction, while most WT cells contain a single focus of Cse4-GFP, no GFP signal is detectable in most *clr4*Δ cells (Fig 5C). The induction time for Cse4-GFP in *clr4*Δ to yield GFP signal in nearly all cells is ~20% longer than in WT (S2A Fig). Western blot analysis confirmed that Cse4-GFP protein level in *clr4*Δ is reduced by at least 50% (Fig 5D). To test the stability of Cse4 in *clr4*Δ cells, we analyzed protein levels of Cse4-GFP over time by western blotting. We observed that after treatment of cycloheximide, Cse4 level decreases faster in *clr4*Δ cells than wild type, suggesting heterochromatin promotes stability of Cse4 in fission yeast (S7 Fig). In addition, we found that the level of Cse4 ubiquitination is significantly enhanced in both *clr4*Δ and *dcr1*Δ by immunoprecipitation assays (Figs 5E, 5F and S8). These data indicate that RNAi and heterochromatin play a vital role in preventing ubiquitin-mediated degradation of Cse4.

Our results predict that if ubiquitin-proteasome pathway is disrupted in the *clr4*Δ mutant, the level of Cse4 will increase; yet due to loss of heterochromatin restriction, Cse4 can incorporate into chromatin but in a more random manner. To test this, we expressed Cse4-GFP in the *clr4*Δ *mts2-1* double mutant. At the 23°C permissive temperature, Cse4-GFP is largely diffused in the nucleus without forming visible foci in *clr4*Δ *mts2-1* (Fig 5G). However, after 4 hours of incubation at the restrictive temperature of 37°C, which blocked proteasome activity, many distinct foci of Cse4-GFP (commonly 6–30) were found and randomly distributed in the nucleus in the double mutant (Fig 5G). The random distribution of Cse4-GFP foci likely results from lacking restriction by defined heterochromatin structure in the double mutant. Western blot assays confirmed that Cse4-GFP level is enhanced in *clr4*Δ *mts2-1* cells at 37°C (S9A Fig). Furthermore, *in situ* chromatin binding assay was performed to test the association of Cse4-GFP foci with chromatin. While diffused Cse4-GFP signal can be readily washed away by Triton-X100 in *clr4*Δ *mts2-1* cells cultured at 23°C, Cse4-GFP foci resulting from proteasome inactivation at 37°C are stably associated with chromatin (S9B Fig). These data support that heterochromatin promotes Cse4 stability and its association with centromere by protecting Cse4 from ubiquitin-dependent proteolysis.

### Heterochromatin promotes centromeric targeting of Cnp1

**Fig 6:**
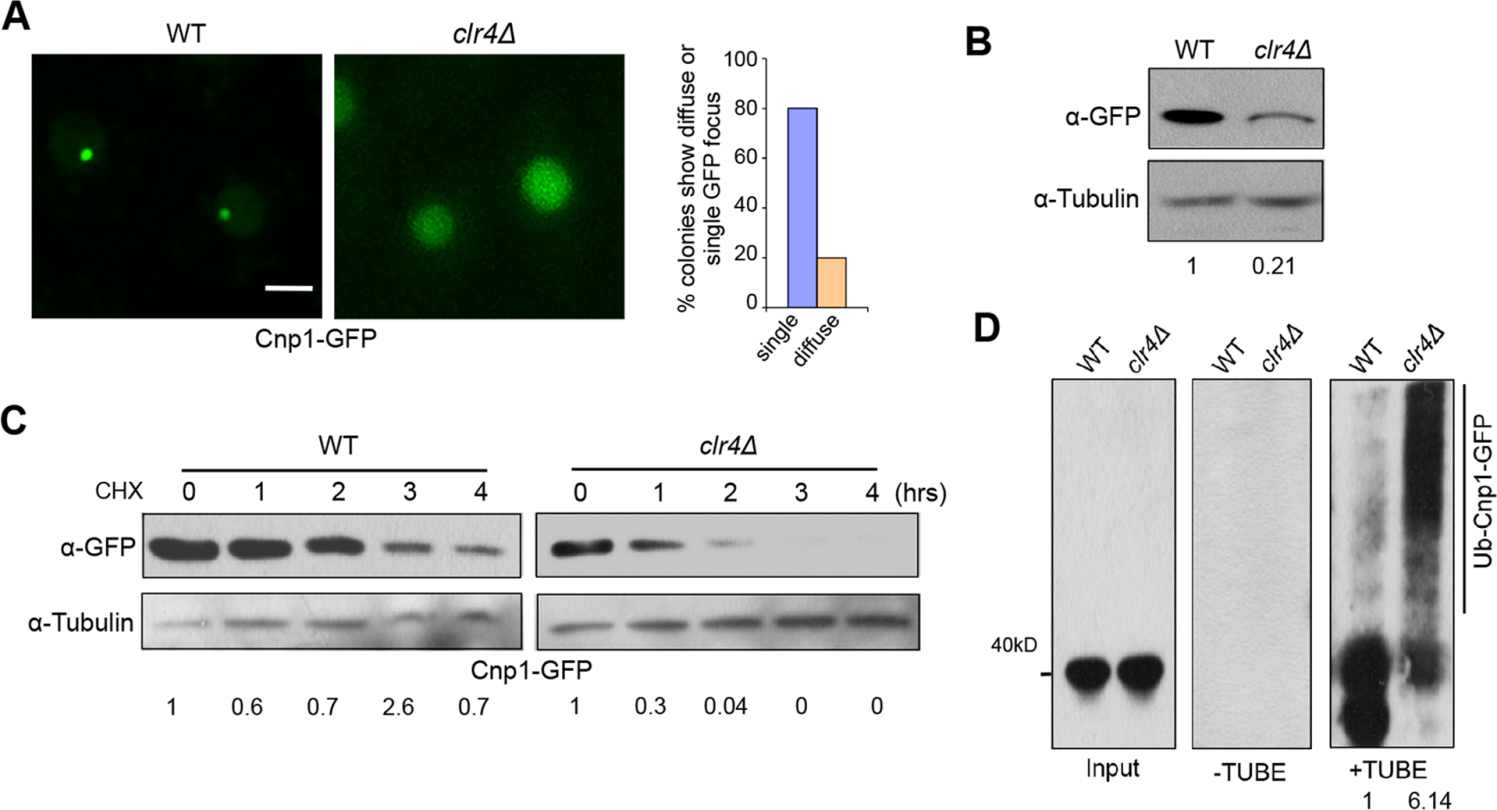
Heterochromatin promotes centromeric targeting of Cnp1 by preventing ubiquitin-mediated degradation. **A,** *clr4*Δ colonies expressing Cnp1-GFP under its native promoter obtained by crossing exhibited a diffuse GFP signal or single focus. (Right) Percentage of colonies displaying diffuse GFP or a single focus, and quantifications were based on random colony analysis. Scale bar: 2 μm. **B,** Western blot analysis of *clr4*Δ cells expressing Cnp1-GFP. WT was used as control. **C,** Stability assays for WT and *clr4*Δ cells expressing Cnp1-GFP. **D,** Extracts from indicated cells were analyzed by TUBE assays. **C,D,** Induction time:20 hours for WT and 22 hours for *clr4*Δ.

Although pericentromeric heterochromatin has been shown to be important for centromere function and identity, a previous study reported that Cnp1-GFP is still associated with centromeres in *clr4* mutant [6]. In light of our findings that centromeric targeting of Cse4 is significantly impaired in *clr4*Δ cells, we tested to what extend Cnp1 distribution is affected by heterochromatin. We examined ~100 colonies generated by genetic cross of *clr4*Δ and cells expressing Cnp1-GFP driven by endogenous promoter. Largely consistent with the previous report, we found that in 80% of the colonies, Cnp1-GFP displays a single focus in *clr4*Δ cells. Remarkably, we also observed that 20% of colonies exhibited faint Cnp1-GFP signal diffused throughout the nucleus (Fig 6A). It is to be noted that the colonies displaying total diffusion will adopt the single-focus phenotype when cultured in liquid rich medium (YES medium), or maintained for generations on the plates. This suggests that the diffused Cnp1-GFP phenotype in clr4Δ cells is not stable, but nonetheless these findings indicate that heterochromatin indeed contributes to the proper localization of Cnp1.

The observation that heterochromatin is not entirely responsible for centromeric localization of Cnp1 suggests that heterochromatin works in parallel with other pathways to ensure proper positioning of CENP-A at centromeres. Consistent with this idea, while Cnp1-GFP in *scm3-19*, a *ts* mutant of the CENP-A chaperone Scm3/HJURP, displays centromeric localization at the permissive temperature 23°C, we found that all colonies of the *clr4*Δ *scm3-19* double mutant that we analyzed contain more than 12% of cells displaying diffused GFP signal at the permissive temperature (S10 Fig). Unlike in *clr4*Δ cells, the diffused phenotype of Cnp1-GFP in the fraction of *clr4*Δ *scm3-19* cells can be observed when cells are cultured on the plate and in the liquid culture, and are persistent over generations. These observations indicate while Cnp1 centromere association is mildly impaired in *clr4*Δ and *scm3-19* single mutant cells, the defect in the double mutant is stronger, suggesting that heterochromatin may function in parallel with Scm3 to protect Cnp1 from degradation.

### Heterochromatin protects native Cnp1 from ubiquitin-mediated degradation

To test whether heterochromatin affects ubiquitin-mediated Cnp1 degradation as observed for Cse4, we first examined the protein level of Cnp1 in *clr4*Δ cells. We found that Cnp1 is significantly lower in *clr4*Δ than in WT (Fig 6B). Consistent with this, we observed that after treatment of cycloheximide, Cnp1 level persists up to 2 hours in wild type cells, while in *clr4*Δ cells, Cnp1 is largely degraded within 1 hour, suggesting heterochromatin promotes stability of Cnp1 in fission yeast (Fig 6C). Next, we performed affinity pull-down using TUBEs with *mts2-1* strains expressing Cnp1-GFP in wild type and *clr4* mutant backgrounds. Upon immunoprecipitation with agarose–TUBEs, we observed a minimal smearing pattern for Cnp1-GFP expressed in wild type cells. However, in *clr4*Δ cells, we found an enhanced smearing pattern in the high molecular mass region, indicative of polyubiquitination (Fig 6D). Similar pattern is observed with the reverse pull-down assay (S11A and S11B Fig). These data support that heterochromatin structure protects native CENP-A from ubiquitin-mediated degradation.

**Fig 7:**
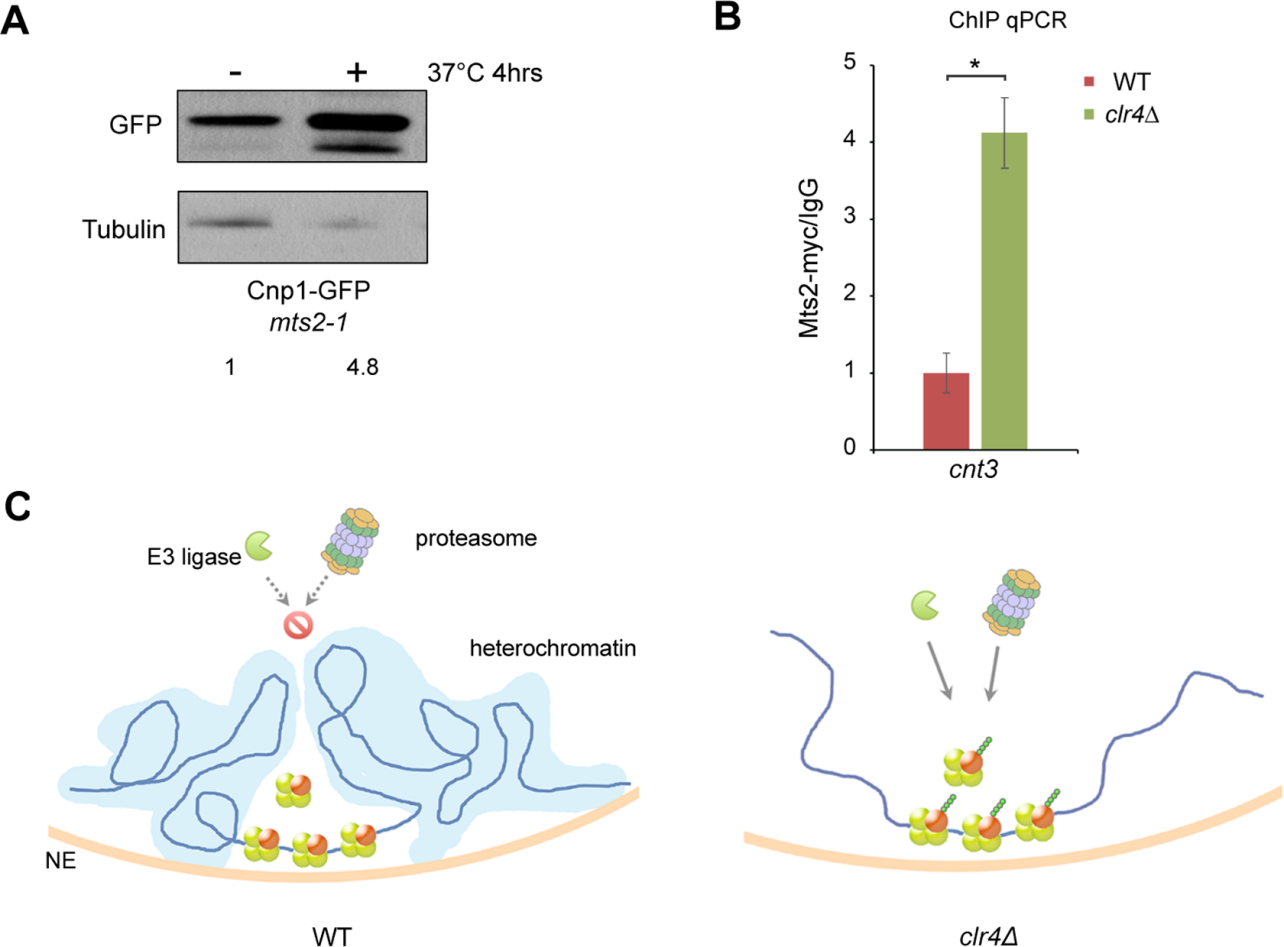
Heterochromatin restricts access of proteasome to centromeric regions. **A,** Cnp1-GFP level is increased in *mts2-1* cells after incubation at 37°C for 4 hours. Western blotting was performed using an anti-GFP antibody. **B,** The association of proteasome with centromeres is increased in *clr4*Δ. ChIP assays were conducted with indicated cells expressing Mts2-myc using an anti-myc antibody. Data from ChIP with the myc antibody were normalized against those from IgG mock ChIP. *cnt3*, centromeric region. n = 8, error bar represents SEM. *, p<0.01. **C,** Model: heterochromatin mediates centromere specificity by blocking the access of ubiquitin-proteosome machinery to centromeres to prevent the degradation of CENP-A.

We speculate that the peri-centrimeric heterochromatin domains may serve as a protective environment to restrict access of proteasome machinery to centromere. This model predicts that proteasome proteins are enriched at the centromere regions in heterochromatin-disrupted cells. We examined the level of myc-tagged Mts2, the19S proteasome subunit, in centromeres by ChIP. We first confirmed that Cnp1 protein level is indeed accumulated in *mts2-1* cells when Mts2 function is blocked by incubation at non-permissive temperature, indicating that the degradation of Cnp1 requires Mts2 (Fig 7A). Our ChIP assays showed that Mts2 is highly enriched in centromeres in *clr4*Δ cells relative to wild type (Fig 7B), demonstrating that heterochromatin is important for preventing the association of proteasome machinery with centromeres. We also found that the level of Mts2 is increased at pericentromere and subtelomeric regions in the absence of heterochromatin (S12 Fig).

## Discussion

One of the most noticeable features of regional centromeres is that they are embedded in heterochromatin [1, 8]. Here we demonstrated that heterochromatin mediates centromere specification by preventing ubiquitin-mediated degradation of CENP-A at centromeres. Our results reveal a previously unrecognized mechanism of heterochromatin in the regulation of centromeres, and represent a significant advance toward our understanding of the interaction between different chromatin domains. This work also provides insights to the dramatic evolutionary transition between regional centromeres and point centromeres.

Although centromere is vital for all eukaryotic organisms studied to date, it is considered as one of the most rapidly evolving sequences of the genome [8, 48]. CENP-A is considered as the epigenetic mark for specifying centromeres [1–3, 49]. How CENP-A is precisely positioned to centromere remains poorly understood. Unlike regional centromeres, budding yeast contains a point centromere defined by the underlying DNA sequence [14, 15]. In this study we expressed budding yeast CENP-A homolog Cse4 in the fission yeast, and found that despite having vastly different N-terminus tails, Cse4 targets to centromere and can at least in part substitute Cnp1. Previous reports have also shown that Cse4 can targets to centromeres when expressed in human cells [50, 51]. These data suggest that the function of CENP-A is largely conserved through evolution. Notably, overexpression of Cse4 leads to its association with heterochromatin, reminiscent of the behavior of overexpressed Cnp1 [37]. However, we found that Cse4 protein level is significantly lower than that of Cnp1 expressed in the same condition. Further experiments demonstrate that Cse4 is subject to efficient ubiquitin-mediated proteolysis, which explain the low level of Cse4 in fission yeast. Cse4 in budding yeast is also strongly regulated by ubiquitin-mediated degradation, and we speculate this which may at least partially explain why overexpression of Cse4 in budding yeast leads to no obvious defects [26, 47].

We showed that deletion of N terminus of Cse4 results in increased level of Cse4 and diffuse nuclear distribution. Our *in vivo* ubiquitination assays indicated that ubiquitination is much reduced in the N-terminus-deleted Cse4. Our domain-swap experiments confirmed that N terminus of Cse4 is largely responsible for its low protein stability. The C-terminus in Cse4 has also been shown to be subject to ubiquitination [30, 52]. It is likely that the C-terminal domain of Cnp1 is also ubiquitinated. Since the domain is highly conserved, the terminus may have minor contribution to the difference in expression level of the two proteins. Although both Cse4 and Cnp1 are subject to ubiquitin-mediated proteolysis, higher level of ubiquitination is observed for Cse4. We speculate that the long N terminus tail of Cse4 may present stronger affinity for E3 ligase, resulting in fast protein turnover rate for Cse4. Together, our results showed that ubiquitination at N-terminus of CENP-A plays an important role in regulating its stability, which in turn governs its proper distribution in centromeres.

Remarkably, we found the centromeric association of Cse4 is completely disrupted when heterochromatin and RNAi are impaired in fission yeast. In addition, we found that Cse4 is expressed at lower level in the heterochromatin mutants relative to wild type. Cse4 in these mutants is less stable and subject to enhanced ubiquitination. These results indicate that heterochromatin acts to protect Cse4 in centromeres from ubiquitin-mediated degradation. The alternative model is that centromere targeting may be affected in *clr4*Δ, resulting in the degradation of soluble pools of CENP-A that become unstable when not bound to chromatin. Although we cannot exclude the possibility, our data do not support this model: we found that Cse4-GFP appears diffused in the *clr4*Δ *mts2-1* double mutant at the permissive temperature, but after blocking proteasome activity at the restrictive temperature, many distinct foci of Cse4-GFP are formed in the nucleus. The association of these Cse4-GFP foci with chromatin in the double mutant appears stable, as shown by our *in situ* chromatin binding assay. Furthermore, our ChIP assays demonstrated the19S proteasome subunit Mts2 is drastically enriched in centromeres in the *clr4*Δ mutant.

This dramatic effect of heterochromatin on Cse4’s stability and localization is unexpected, given that previous reports showed that Cnp1 remains associated with centromere in the absence of heterochromatin [6, 9]. Our closer examinations showed that some *clr4*Δ colonies indeed display faint, diffuse Cnp1-GFP signal in the nucleus, indicative of unstable association of Cnp1 with centromeres. We further found that in *clr4*Δ cells Cnp1 has reduced protein level, faster turnover rate and higher ubiquitination, similar to Cse4 in the mutant. Heterochromatin thus also protects Cnp1 from ubiquitin-mediated degradation. Consistent with our work, it has been shown that the 19S proteasome subunit Rpt3 also plays an important role in distribution of CENP-A [53]. Nevertheless, we cannot exclude the possibility that non-proteolytic functions of 19S proteasome may participate in the regulation of CENP-A positioning.

The mild Cnp1 distribution defects in heterochromatin mutants suggest that redundant pathways may be involved in preventing Cnp1 degradation in centromeres. Indeed, we found that the *clr4*Δ *scm3-19* double mutant showed the synthetic defects in Cnp1-GFP distribution. Scm3/HJURP is a conserved CENP-A chaperone that has been implicated in protecting Cse4 degradation in budding yeast [27, 54]. On the other hand, the centromere association of Cse4 in fission yeast strongly depends on heterochromatin. It is possible that exogenous Cse4 is unable to efficiently utilize the other CENP-A protection pathway, such as Scm3, in fission yeast. The higher turnover rate of Cse4 may also contribute to its strong dependence on heterochromatin protection.

The heterochromatin-mediated safeguard mechanism for centromeres may help explain the long-standing puzzle of why centromeres are usually flanked by heterochromatin [1]. It may also provide at least partial explanation for the observation that Cse4 can localize to centromeres in HeLa cells [50, 51]. Previous studies have shown that overexpression of CENP-A in fission yeast and *Drosophila* results in specific enrichment of CENP-A in heterochromatin [19, 35, 37]. CENP-A from budding yeast, *Caenorhabditis elegans* and human expressed in *Drosophila* is preferentially localized at pericentromeric heterochromatin [50]. Neocentromeres are often formed in heterochromatic regions in a variety of organisms [5, 55–60]. In an especially dramatic case, a heterochromatin block without native centromeres can exhibit centromere activity in *Drosophila* [61]. Heterochromatin is also important for centromere localization of CENP-A in *Neurospora crassa* and mouse cell lines [62, 63]. We suggest that protection of CENP-A from degradation by heterochromatin may be a common mechanism used for centromere assembly.

How heterochromatin protects CENP-A from ubiquitin-mediated degradation remains to be elucidated. Using a repeat-specific reporter, we recently showed that the tandem arrays at pericentromeric heterochromatin in fission yeast are organized into a specific three-dimensional architecture [7]. We propose that pericentromeric heterochromatin forms a distinct higher-order structure that restricts the access of ubiquitin-proteasome machinery to centromeres, which in turn avoids degradation of CENP-A in the region (Fig 7C). The unique spatial architecture of pericentromeric heterochromatin may account for why CENP-A prefers this region rather than other heterochromatin regions.

How the dramatic evolutionary transition between regional centromeres and point centromeres occurred is a fascinating but unsolved mystery in centromere evolution [8]. The gaining of point centromere and the loss of heterochromatin and RNAi machinery appear to occur concomitantly during the evolution [8]. We speculate that lacking the protection of Cse4 by pericentromeric heterochromatin in budding yeast may contribute to the arising of small, compact “point” centromeres and the adaptation of the centromeres to being genetically defined. For small “point” centromeres, it is possible that the kinetochore complex and Scm3 can provide sufficient protection for centromeric Cse4 from degradation [26, 27, 32]. But large regional centromeres may require additional mechanisms, such as peri-centromeric heterochromatin, to prevent ubiquitin-mediated proteolysis of CENP-A at centromeres. We also noted that not all neocentromeres are assembled in or near heterochromatin [64, 65], suggesting that mechanisms other than heterochromatin are involved in neocentromere formation. It will be interesting in future studies to identify these mechanisms, which may provide important new insights into centromere specification and evolution.

## Materials and methods

### Strains, media, and DNA constructs

Standard media and genetic analysis for fission yeast were used [66]. Cse4, Cnp1, Cse4-NΔ, and domain-swap constructs were cloned into the pREP1 vector under the *nmt1* promoter in frame with GFP. Fission yeast strains used in this study are listed in the Supporting Information S1 Table.

### *in situ* chromatin-binding assay

*in situ* chromatin-binding assays were performed as described previously [37, 67]. Briefly, log-phase cells were collected and incubated at 32°C in ZM buffer (50 mM sodium citrate pH 5.6, 1.2 M sorbitol, 0.5 mM MgAc, 10 mM dithiothreitol, 2 mg/ml Zymolase) for 30 min. After centrifugation, cells were then washed twice with STOP buffer (0.1 M MES pH 6.4, 1.2 M sorbitol, 1 mM EDTA, 0.5 mM MgAc). Cells were resuspended with EB buffer (20 mM PIPES–potassium hydroxide, pH 6.8, 0.4 M sorbitol, 2 mM MgAc, 150 mM KAc) ±1% Triton X-100 at room temperature for 7 min. Following fixation with 3.7% formaldehyde and 10% methanol, cells were examined by DeltaVision imaging system.

### Microscopy

Cells were imaged using the DeltaVision System (Applied Precision, Issaquah, WA). Images were taken as z-stacks of 0.2-μm increments with an oil immersion objective (×100). Standard DAPI staining and analysis methods for fission yeast nuclei were used.

### Immunoprecipitation

Exponentially growing *mts2-1* cells carrying indicated pREP1 construct were induced in minimum media without thiamine. After inductions were confirmed by visualizing GFP signal under microscope, cells were incubated in 37°C for 4–6 hrs to inactivate proteasome. Cell lysates were prepared in 1x lysis buffer supplemented with 20 μM MG132 (Selleck Biochemicals), 20 μM PR-619 (LifeSensors), 5 μM 1,10-phenanthroline (LifeSensors), 10 μM PMSF and protease inhibitor cocktail (Sigma-Aldrich). Cell extracts were then incubated with an anti-GFP antibody (Santa Cruz, sc-9996) overnight at 4°C. IgG-coated magnetic beads (ThermoFisher Scientific) were added, followed by incubation at 4°C for 2 hrs. After immunoprecipitation, beads were washed 3 times using lysis buffer. Proteins were eluted and analyzed by western blotting using an anti-pan-ubiquitin (Cell Signaling, P4D1) antibody.

### Ubiquitin affinity pull-down assays

Ubiquitinated proteins were pulled down using Tandem Ubiquitin Binding Entities (TUBEs) (LifeSensors) according to the manufacturer’s instructions. Briefly, protein extracts prepared as described above were incubated with TUBE agarose or empty agarose beads overnight at 4°C. The bound proteins were eluted from washed beads and analyzed by western blotting using an anti-GFP antibody (Santa Cruz, sc-9996).

### Western blot analysis

Cell extracts from log-phase cells were prepared using standard protocols. Extracted proteins were separated on SDS-polyacrylamide gels and blotted onto PVDF membranes. Blots were probed with anti-GFP (Santa Cruz, sc-9996), anti-tubulin (Abcam, ab6160), or anti-pan-ubiquitin (Cell Signaling, P4D1) antibodies.

### ChIP

ChIP assays were carried out as described [43]. Briefly, cells were grown to log phase at 30°C, and cross-linked by treatment with 1% formaldehyde for 30 mins with gentle shaking at room temperature. Immunoprecipitation was performed with protein A agarose (KPL) conjugated to the anti-myc antibody (ab32, abcam) and anti-GFP antibody (ab290, abcam). Precipitated DNA was cleaned by MiniElute PCR purification Kit (Qiagen). Two microliters of ChIP or WCE samples were analyzed by quantitative PCR using primers specific to centromeric core region *cnt3*, pericentromeric regions *otr1*, and sub-telomeric regions *subT*. *act1*^+^ was used as the control gene.

### Protein stability assays

Protein stability assays were performed as described [37]. Briefly, after cells were induced in minimum media without thiamine, cycloheximide was added to a final concentration of 100 μg/ml. Lysates from cells collected at the indicated time points were prepared, and analyzed by western blotting using anti-GFP (Santa Cruz, sc-9996) and anti-tubulin (Abcam, ab6160) antibodies. The “0” time point refers to samples taken immediately after cycloheximide was added.

### RT-PCR

RT-PCR assays were performed as described [68]. Briefly, total RNA from log-phase cells was extracted using TRizol. After treatment with DNase I (Promega), ~50 ng of RNA were analyzed using a One-Step RT-PCR kit (Qiagen) with primers specific for GFP. *act1*^+^ was used as an internal control.

## Supporting information

S1 Fig (Related to Fig 1) ChIP qPCR showing relative enrichment of overexpressed Cnp1-GFP in heterochromatin. Centromere: centromeric region (*cnt3*), Otr: peri-centromeric region, subT: sub-telomeric region. ChIP was repeated in triplicate. Error bar indicates SEM.
(TIF)

S2 Fig (Related to Figs 1, 2, 3 and 6) Induction time for pREP1-Cse4-GFP and pREP1-Cnp1-GFP in WT and *clr4*Δ to yield florescent signal in nearly all cells. A) The induction time for indicated protein expression was measured in hours. B) The mRNA expression levels of the indicated genes after induction were measured by qPCR at indicated time points. The expression levels were normalized to those measured at 20hrs time points. The experiment was repeated four times.
(TIF)

S3 Fig (Related to Fig 5) Distribution pattern of Cse4-GFP at the restrictive temperature. Cse4-GFP *mts2-1* cells were induced for 40 hours at 23ºC, then cultured for additional 4 hours at either 23 ºC or 37 ºC. Scale bar: 2μm.
(TIF)

S4 Fig (Related to Fig 4) Sequence alignment of Cse4 and Cnp1. Cse4 and Cnp1 shares conserved C terminus sequence, but very different N terminus tail. Note that Cse4 N terminus tail is much longer than Cnp1. Consensus Centromere Targeting Domain (CATD) was also highlighted.
(TIF)

S5 Fig (Related to Fig 4) The N-terminal domain of Cse4 is important for ubiquitin-mediated Cse4 degradation in fission yeast. **A,** Distribution pattern of Cse4-N**Δ**-GFP after 22-hour induction. **B,** western blot analysis of cells expressing indicated proteins using an anti-GFP antibody. Tubulin was used as a loading control. **C,** Lysates from cells expressing indicated proteins collected at indicated time points (hrs) following the treatment with cycloheximide were analyzed by western blotting with a GFP antibody. **D,** Extracts from cells expressing indicated proteins were subject to immunoprecipitation with an anti-GFP antibody. Precipitates were analyzed by western blotting using an anti-ubiquitin antibody. N**Δ**-GFP, Cse4-N**Δ**-GFP. Induction time for all the strains used in A-D was 24 hours.
(TIF)

S6 Fig (Related to Fig 5) Cse4-GFP images are displayed with same exposure settings to show the difference of signal intensities in wild type and heterochromatin mutants. **A,** Fluorescent images of Cse4-GFP were processed to use exposure setting 1 or 2. In each row, with same exposure settings signal intensity can be compared. **B,** Quantification of the nuclear GFP signal intensity using exposure setting 1. n = 17. Error bar: SEM.
(TIF)

S7 Fig (Related to Fig 5) Cse4-GFP stability is decreased when heterochromatin is impaired. Lysates from cells expressing indicated proteins collected at indicated time points (hrs) following the treatment with cycloheximide were analyzed by western blotting with an GFP antibody. Induction time prior the cycloheximide treatment was 24 hours for Cse4-GFP and 28 hours for Cse4-GFP *clr4***Δ**.
(TIF)

S8 Fig (Related to Fig 5) Ubiquitination level of Cse4-GFP is significantly enhanced in *clr4*Δ and *dcr1*Δ mutant. Extracts from indicated cells expressing Cse4-GFP were subject to immunoprecipitation with an anti-GFP antibody. Precipitates were analyzed by western blotting using an anti-ubiquitin antibody. See Fig 5E and 5F for the input.
(TIF)

S9 Fig (Related to Fig 5) Heterochromatin protects Cse4 from ubiquitin-mediated degradation. **A,** Cse4 level in *clr4*Δ mutant is increased after proteasome inactivation. Cells overexpressing Cse4-GFP in the *clr4***Δ** *mts2-1* double mutant and the single *clr4***Δ** mutant as a control were incubated at 37°C for 4 hours, and were subject to western blot analysis using an anti-GFP antibody. Prior to 37 ºC culture, expressions of Cse4-GFP were induced for 44 hours at 23 ºC. Tubulin was used as a loading control. **B,** *in situ* chromatin-binding assay for the *clr4***Δ** *mts2-1* double mutant cells overexpressing Cse4-GFP. The mutant cells were induced for 44 hours, and then incubated at 37°C for 4 hours before being collected. After washing with Triton X-100, multiple Cse4-GFP foci remained in the nucleus, indicating that Cse4-GFP associates with chromatin.
(TIF)

S10 Fig (Related to Fig 6) Distribution pattern of Cnp1-GFP in the *scm3-19 clr4*Δ double mutant at 23°C. Cnp1-GFP was expressed under the control of its native promoter (integrated in the *ade6* locus). The *scm3-19* mutant expressing Cnp1-GFP at 23°C was used as control. The percentage of cells exhibiting diffuse GFP pattern is indicated at the right.
(TIF)

S11 Fig (Related to Fig 6) Ubiquitination level of Cnp1-GFP is significantly enhanced in *clr4*Δ mutant. **A.** Extracts from indicated cells expressing Cnp1-GFP were subject to immunoprecipitation with an anti-GFP antibody. Precipitates were analyzed by western blotting using an anti-ubiquitin antibody. See Fig 6D for the input. **B**. A biological replicate of the pull down experiment.
(TIF)

S12 Fig (Related to Fig 7) Mst2 is increased in pericentromeric and sub-telomeric regions in *clr4*Δ. ChIP assays were conducted with indicated cells expressing Mts2-myc using an anti-myc antibody. Data from ChIP with the myc antibody were normalized against those from IgG mock ChIP. *otr*, pericentromeric region, *subT*, sub-telomeric region. n = 3, error bar represents SEM. *, p<0.01.
(TIF)

S1 Table Strains used in this study. (DOCX)

S2 Table Primers used in this study. (DOCX)
