# Supplementary figures and images for: Heterochromatin and RNAi regulate centromeres by protecting CENP-A from ubiquitin-mediated degradation
Source: PLoS Genet. 2018 Aug 8;14(8):e1007572. doi: 10.1371/journal.pgen.1007572 (PMC6101405; doi:10.1371/journal.pgen.1007572)

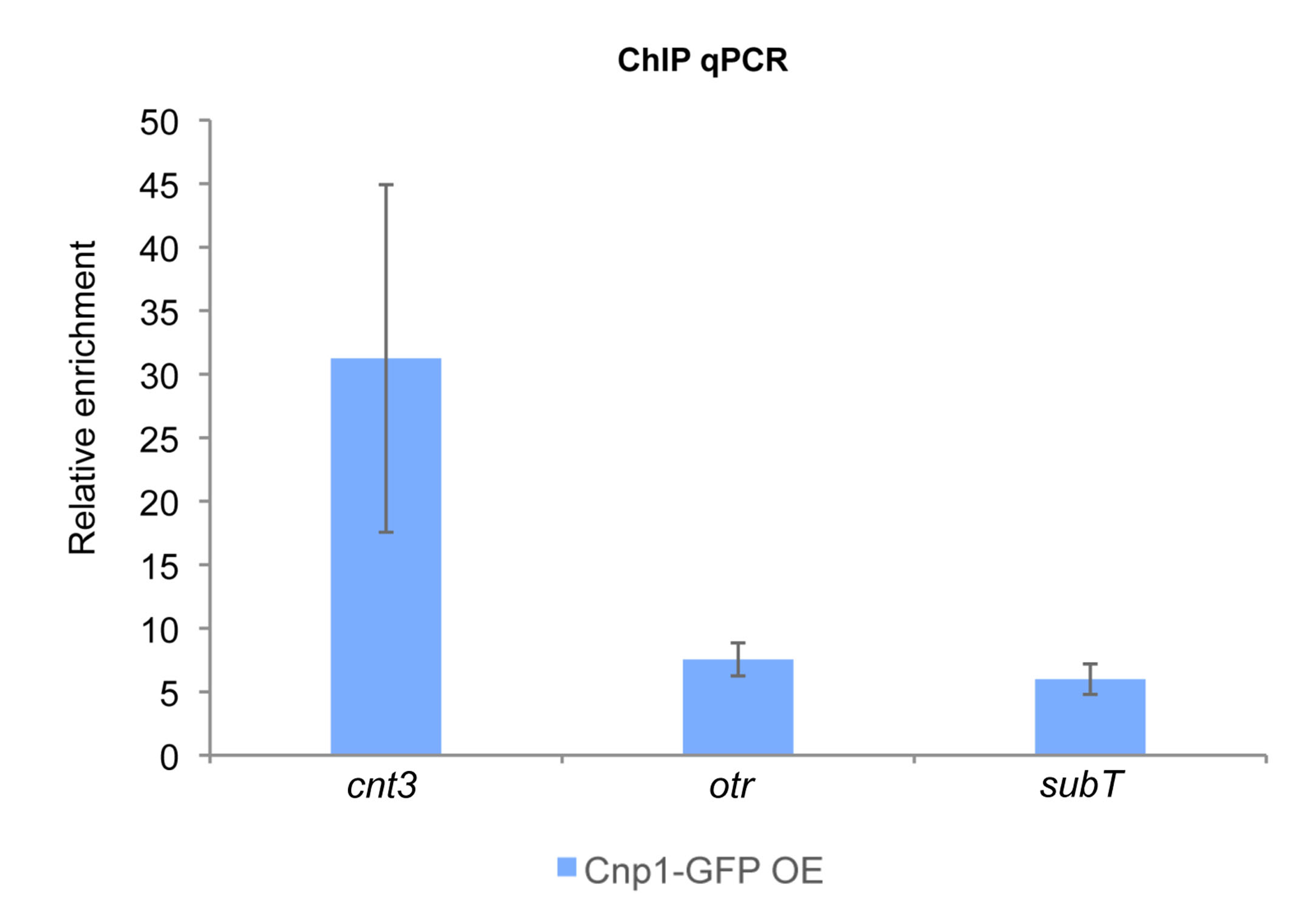

Supplement: S1 Fig — Centromere: centromeric region (cnt3), Otr: peri-centromeric region, subT: sub-telomeric region. ChIP was repeated in triplicate. Error bar indicates SEM. (TIF) [file pgen.1007572.s001.tif]

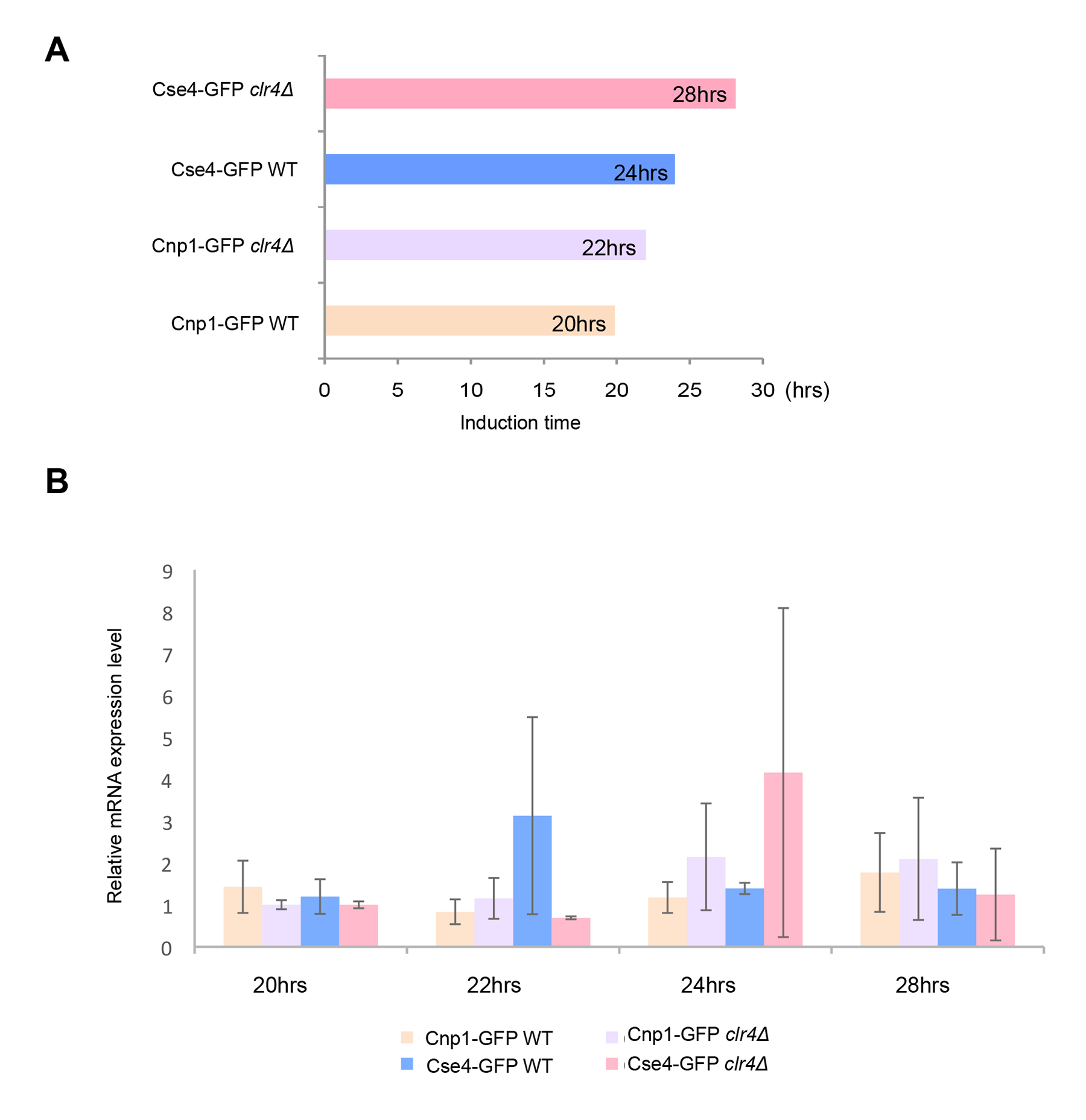

Supplement: S2 Fig — A) The induction time for indicated protein expression was measured in hours. B) The mRNA expression levels of the indicated genes after induction were measured by qPCR at indicated time points. The expression levels were normalized to those measured at 20hrs time points. The experiment was repeated four times. (TIF) [file pgen.1007572.s002.tif]

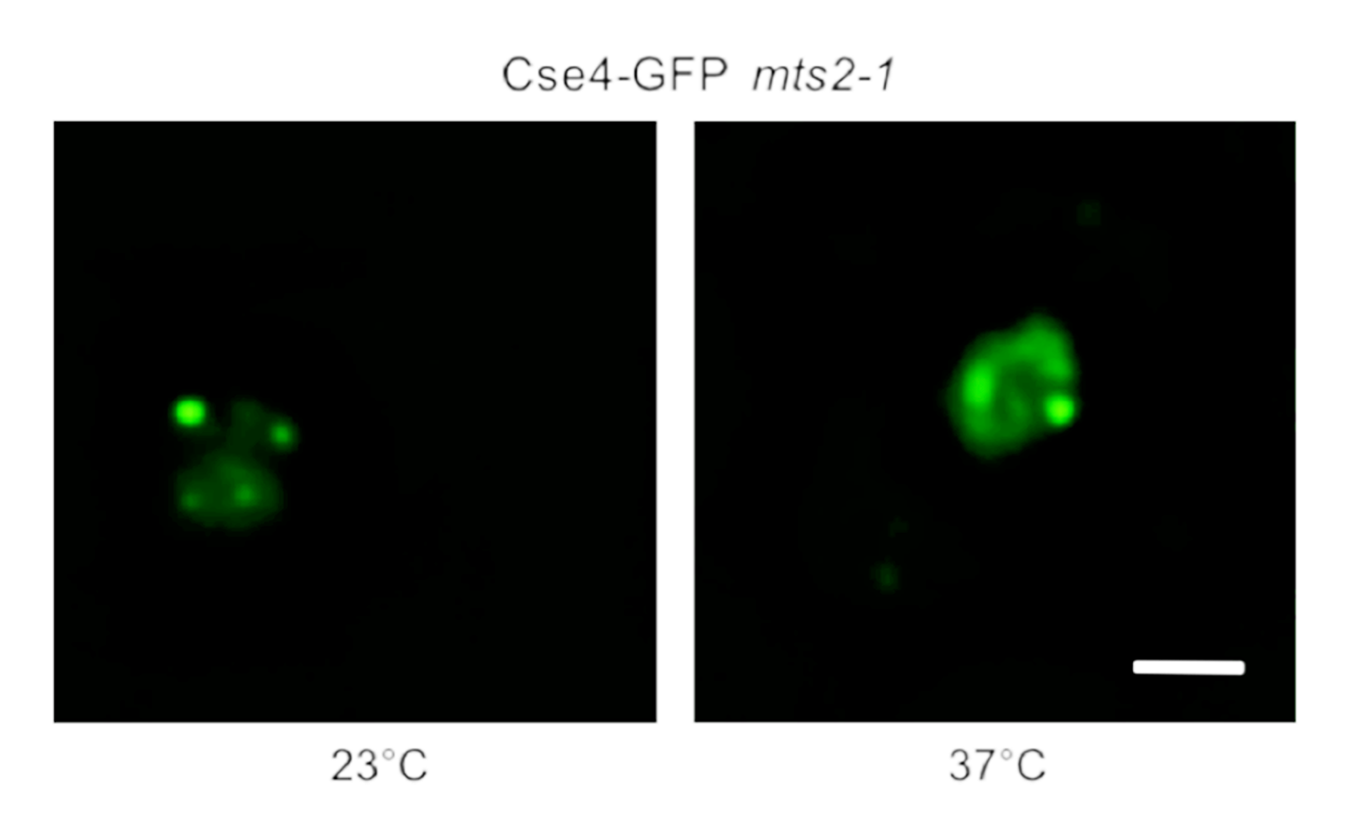

Supplement: S3 Fig — Cse4-GFP mts2-1 cells were induced for 40 hours at 23ºC, then cultured for additional 4 hours at either 23 ºC or 37 ºC. Scale bar: 2μm. (TIF) [file pgen.1007572.s003.tif]

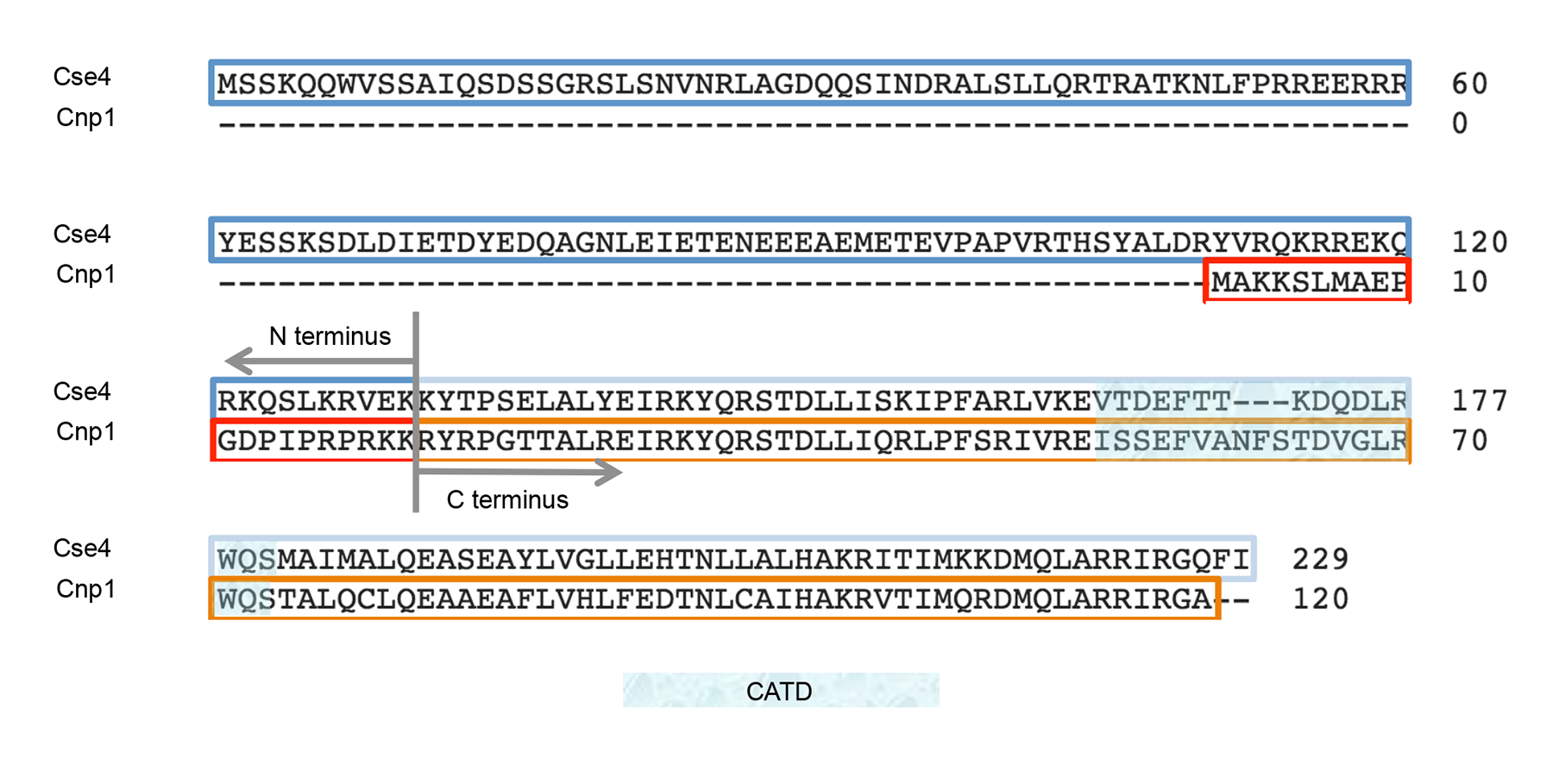

Supplement: S4 Fig — Cse4 and Cnp1 shares conserved C terminus sequence, but very different N terminus tail. Note that Cse4 N terminus tail is much longer than Cnp1. Consensus Centromere Targeting Domain (CATD) was also highlighted. (TIF) [file pgen.1007572.s004.tif]

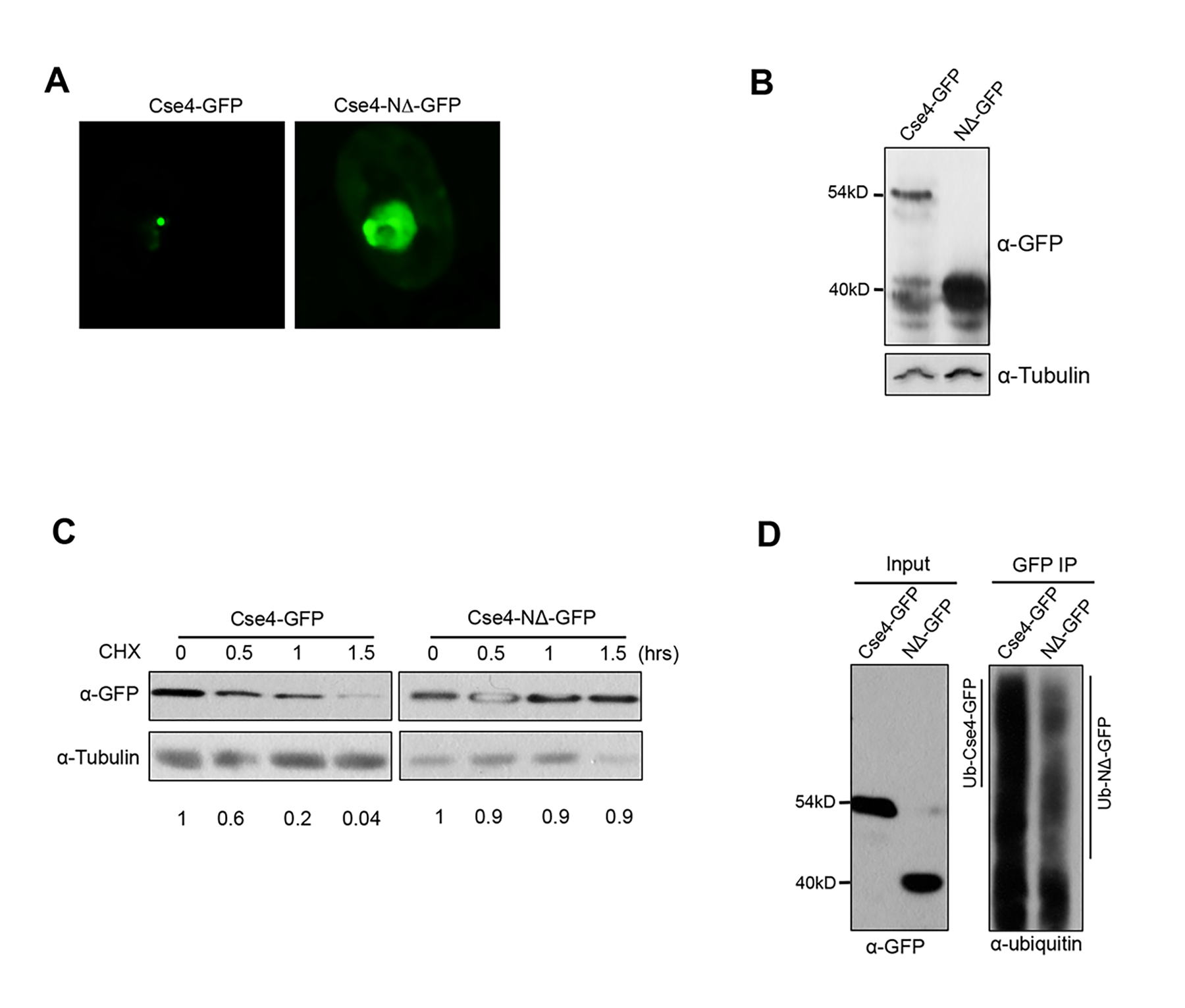

Supplement: S5 Fig — A, Distribution pattern of Cse4-NΔ-GFP after 22-hour induction. B, western blot analysis of cells expressing indicated proteins using an anti-GFP antibody. Tubulin was used as a loading control. C, Lysates from cells expressing indicated proteins collected at indicated time points (hrs) following the treatment with cycloheximide were analyzed by western blotting with a GFP antibody. D, Extracts from cells expressing indicated proteins were subject to immunoprecipitation with an anti-GFP antibody. Precipitates were analyzed by western blotting using an anti-ubiquitin antibody. NΔ-GFP, Cse4-NΔ-GFP. Induction time for all the strains used in A-D was 24 hours. (TIF) [file pgen.1007572.s005.tif]

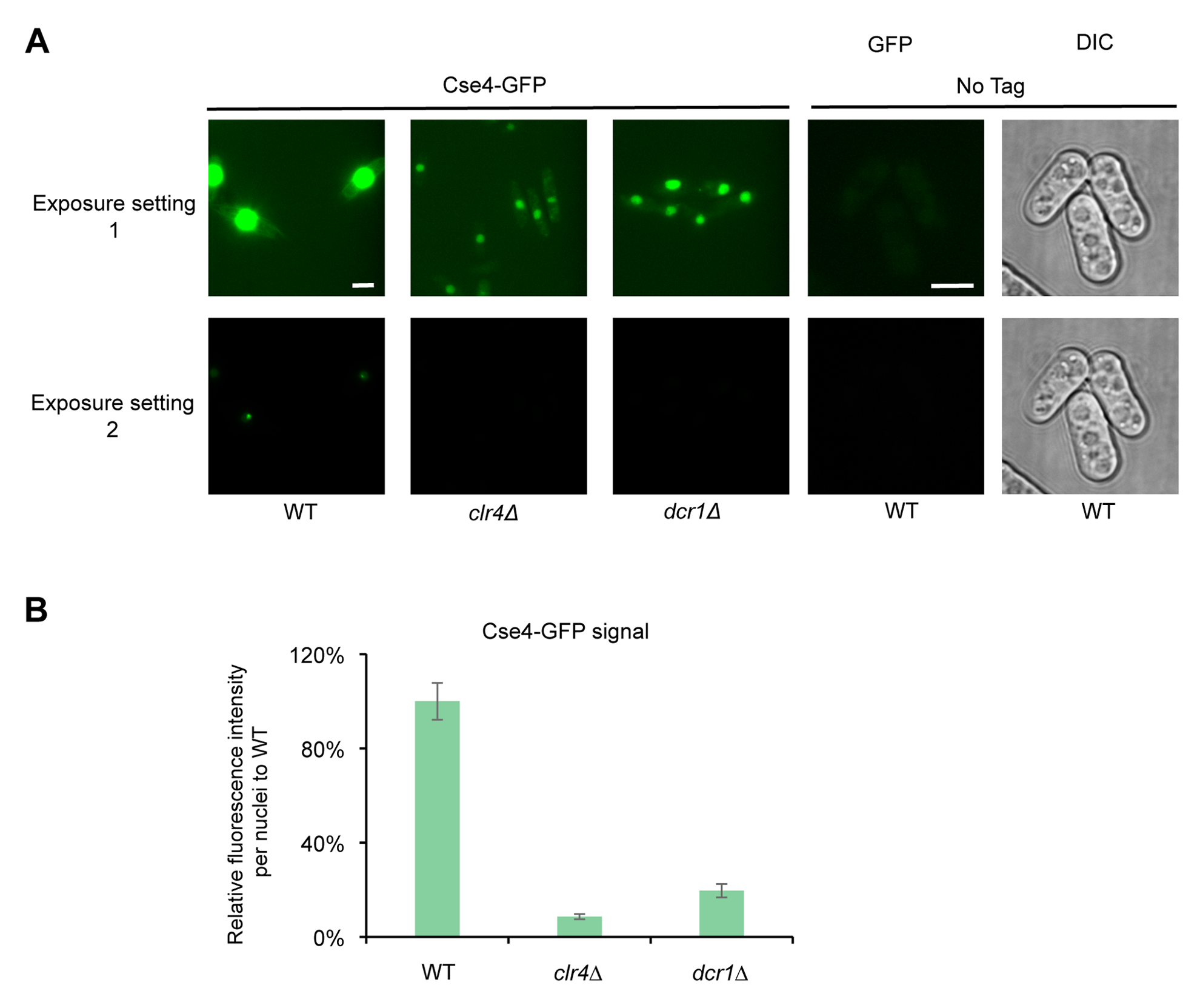

Supplement: S6 Fig — A, Fluorescent images of Cse4-GFP were processed to use exposure setting 1 or 2. In each row, with same exposure settings signal intensity can be compared. B, Quantification of the nuclear GFP signal intensity using exposure setting 1. n = 17. Error bar: SEM. (TIF) [file pgen.1007572.s006.tif]

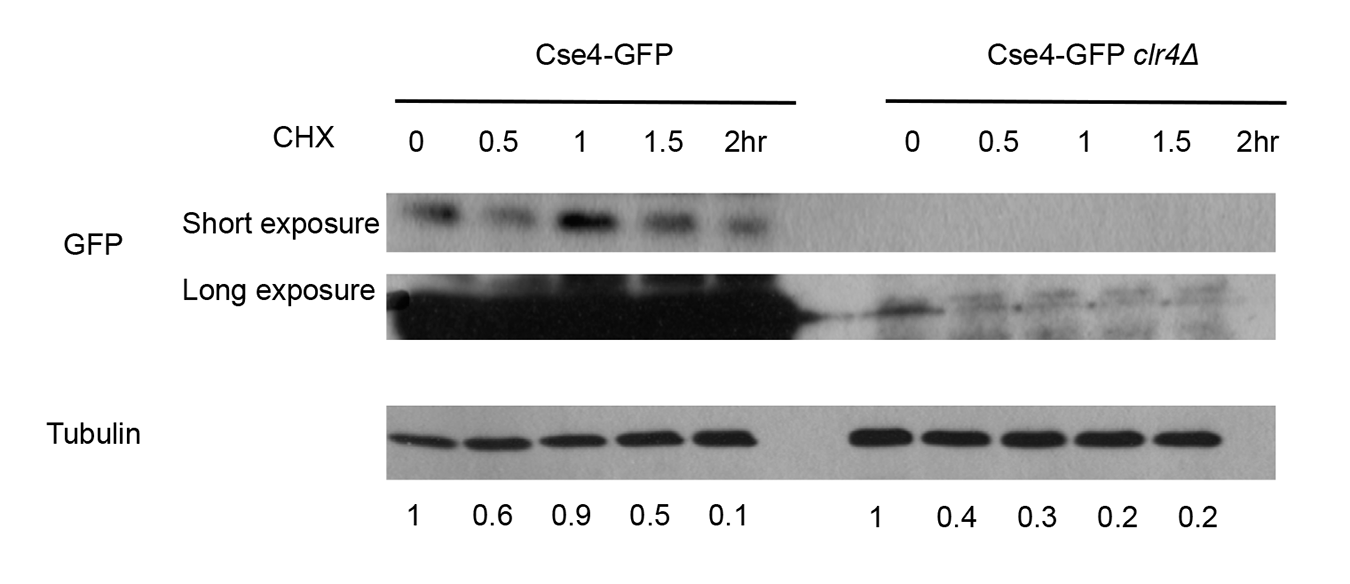

Supplement: S7 Fig — Lysates from cells expressing indicated proteins collected at indicated time points (hrs) following the treatment with cycloheximide were analyzed by western blotting with an GFP antibody. Induction time prior the cycloheximide treatment was 24 hours for Cse4-GFP and 28 hours for Cse4-GFP clr4Δ. (TIF) [file pgen.1007572.s007.tif]

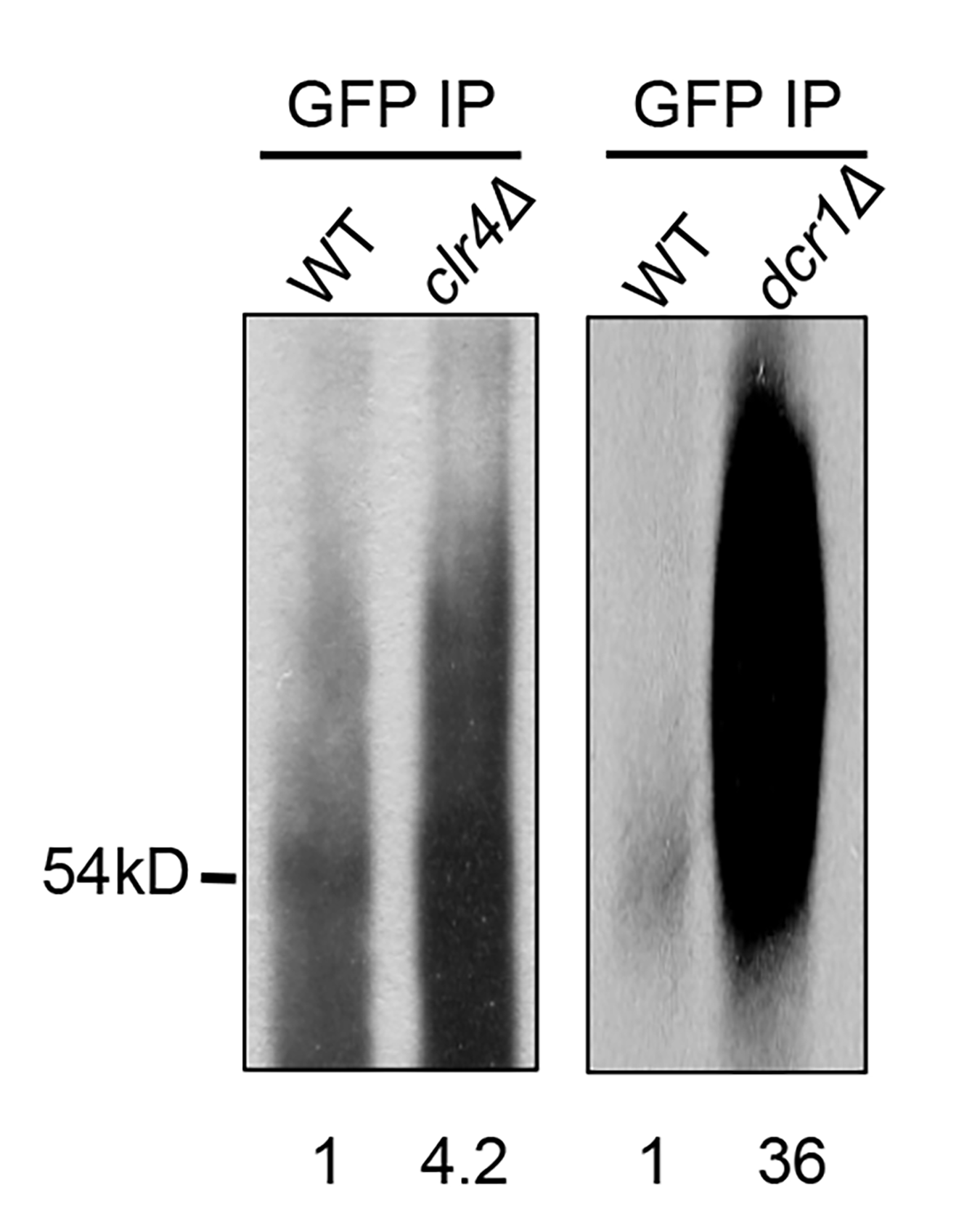

Supplement: S8 Fig — Extracts from indicated cells expressing Cse4-GFP were subject to immunoprecipitation with an anti-GFP antibody. Precipitates were analyzed by western blotting using an anti-ubiquitin antibody. See Fig 5E and 5F for the input. (TIF) [file pgen.1007572.s008.tif]

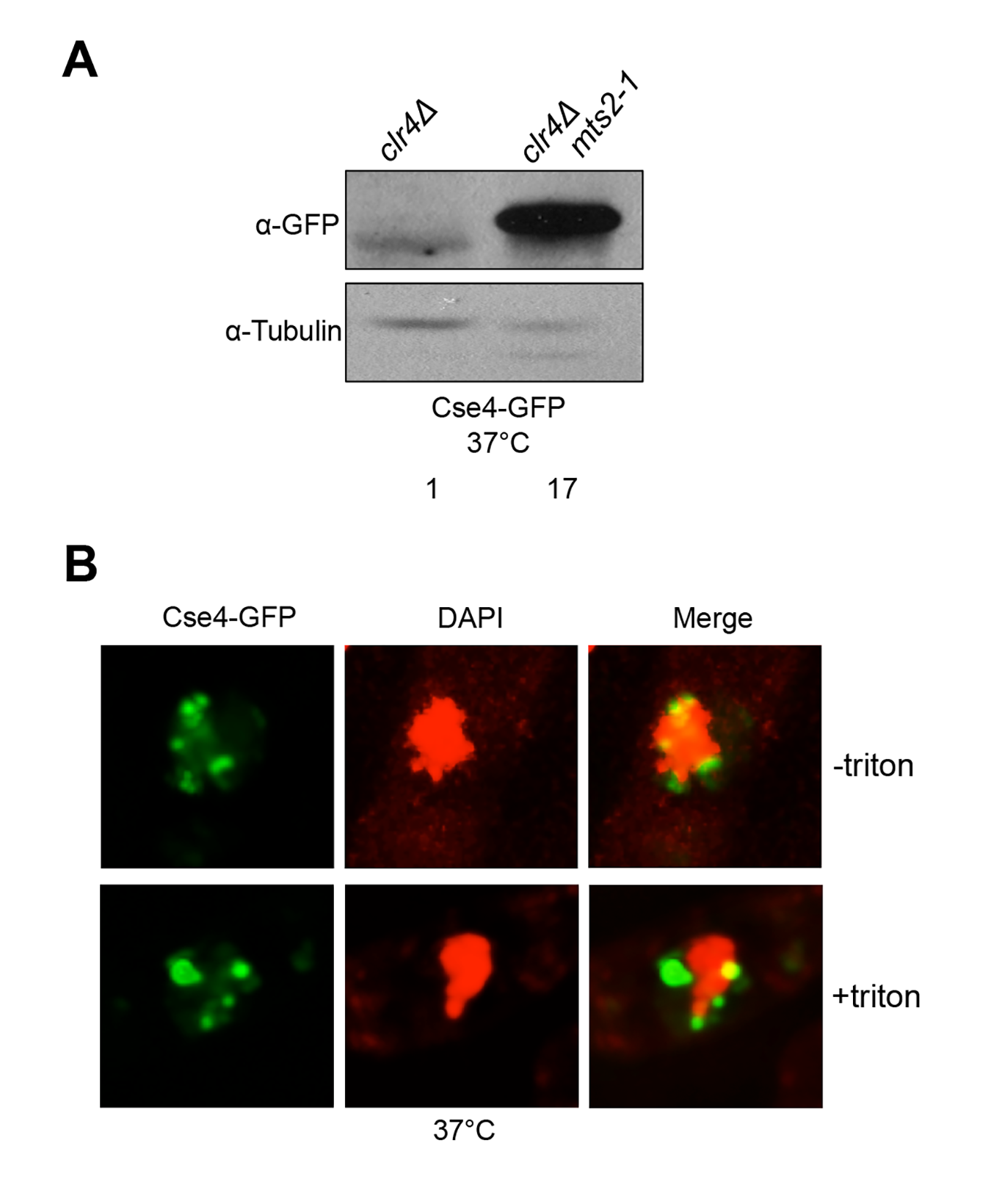

Supplement: S9 Fig — A, Cse4 level in clr4Δ mutant is increased after proteasome inactivation. Cells overexpressing Cse4-GFP in the clr4Δ mts2-1 double mutant and the single clr4Δ mutant as a control were incubated at 37°C for 4 hours, and were subject to western blot analysis using an anti-GFP antibody. Prior to 37 ºC culture, expressions of Cse4-GFP were induced for 44 hours at 23 ºC. Tubulin was used as a loading control. B, in situ chromatin-binding assay for the clr4Δ mts2-1 double mutant cells overexpressing Cse4-GFP. The mutant cells were induced for 44 hours, and then incubated at 37°C for 4 hours before being collected. After washing with Triton X-100, multiple Cse4-GFP foci remained in the nucleus, indicating that Cse4-GFP associates with chromatin. (TIF) [file pgen.1007572.s009.tif]

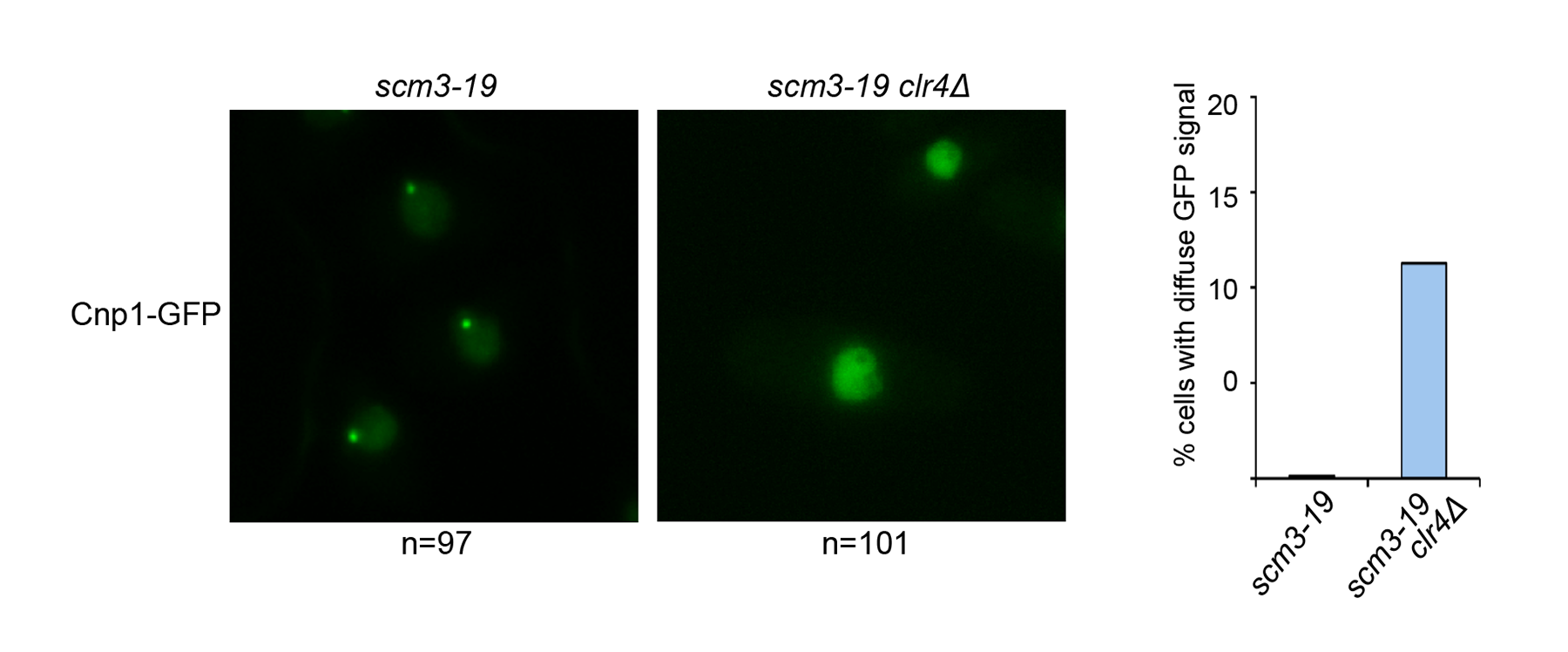

Supplement: S10 Fig — Cnp1-GFP was expressed under the control of its native promoter (integrated in the ade6 locus). The scm3-19 mutant expressing Cnp1-GFP at 23°C was used as control. The percentage of cells exhibiting diffuse GFP pattern is indicated at the right. (TIF) [file pgen.1007572.s010.tif]

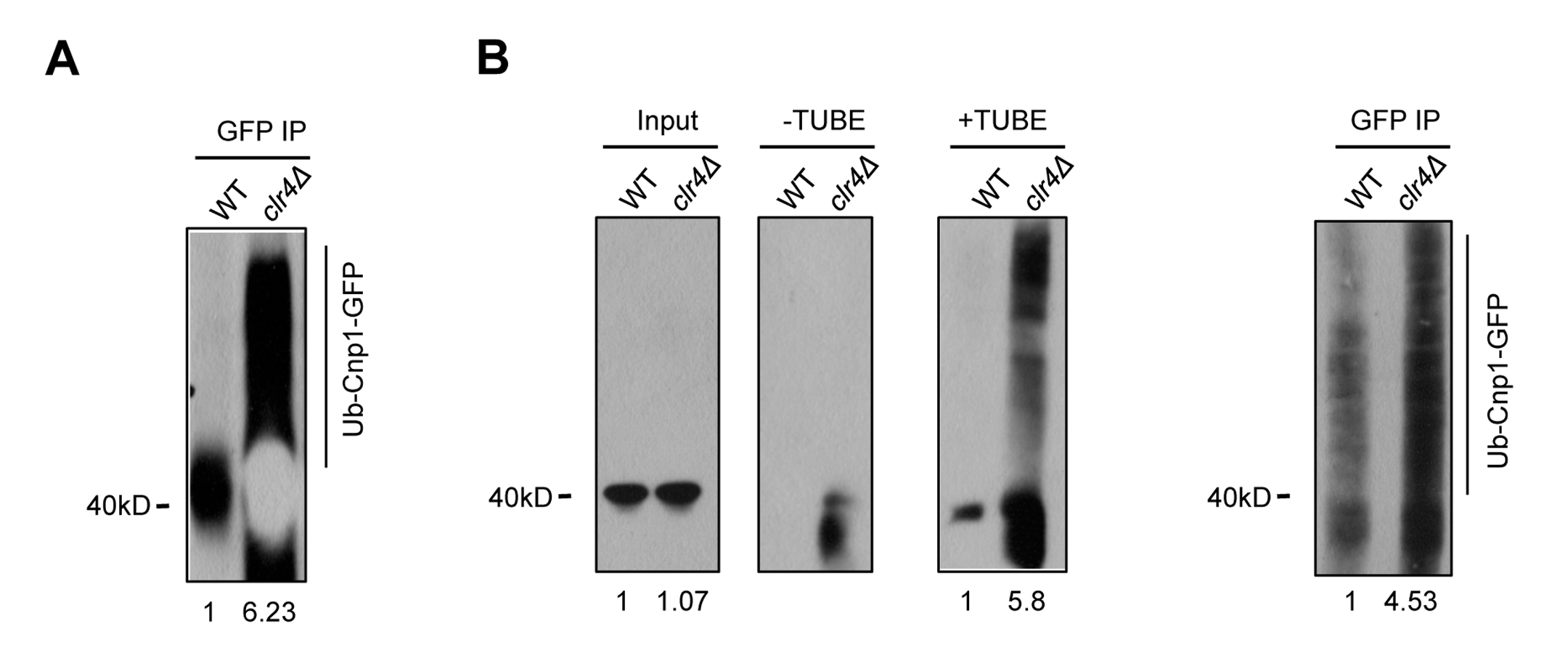

Supplement: S11 Fig — A. Extracts from indicated cells expressing Cnp1-GFP were subject to immunoprecipitation with an anti-GFP antibody. Precipitates were analyzed by western blotting using an anti-ubiquitin antibody. See Fig 6D for the input. B. A biological replicate of the pull down experiment. (TIF) [file pgen.1007572.s011.tif]

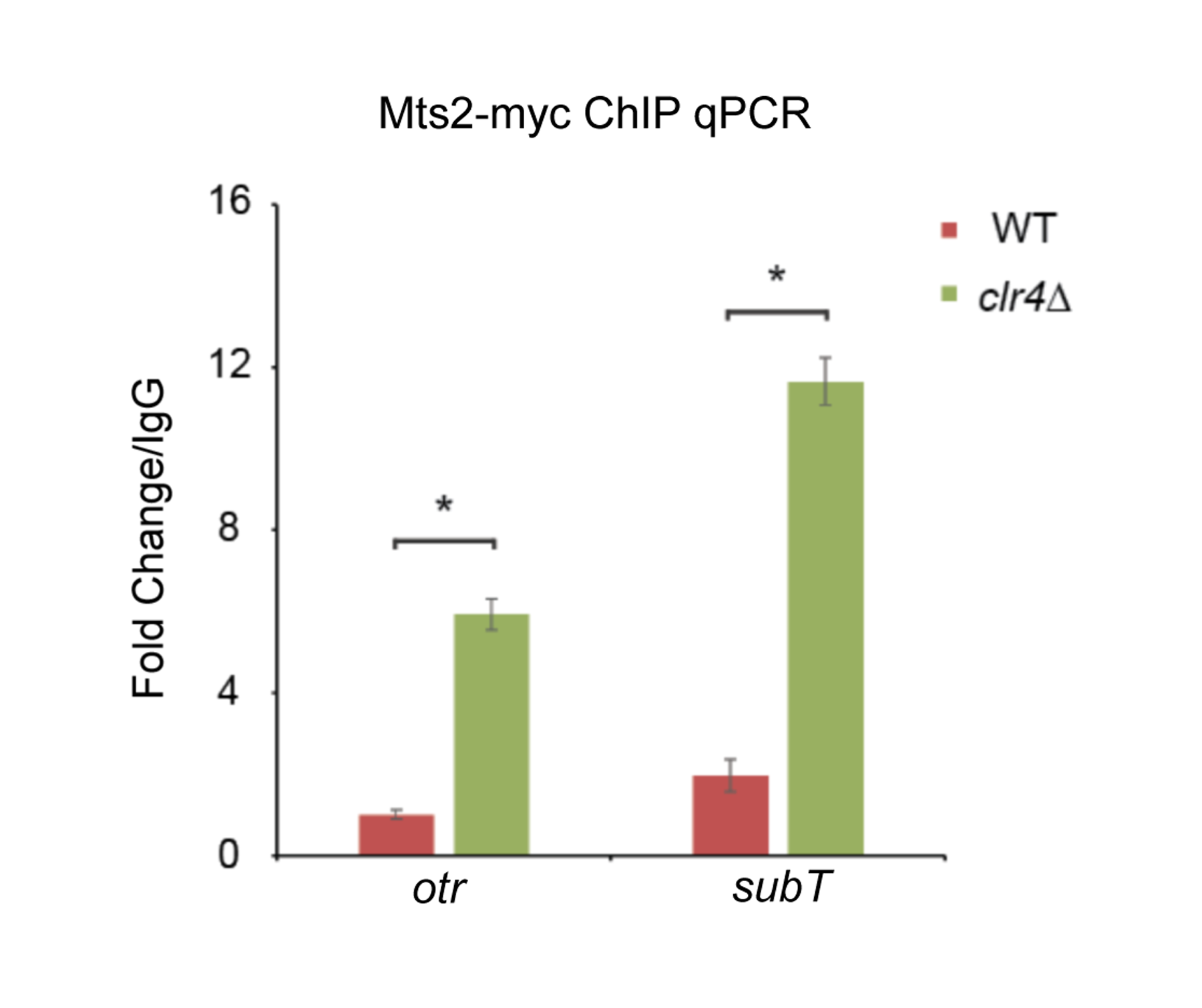

Supplement: S12 Fig — ChIP assays were conducted with indicated cells expressing Mts2-myc using an anti-myc antibody. Data from ChIP with the myc antibody were normalized against those from IgG mock ChIP. otr, pericentromeric region, subT, sub-telomeric region. n = 3, error bar represents SEM. *, p<0.01. (TIF) [file pgen.1007572.s012.tif]
