# Supplementary material for: Heterochromatin and RNAi regulate centromeres by protecting CENP-A from ubiquitin-mediated degradation
Source: PLoS Genet. 2018 Aug 8;14(8):e1007572. doi: 10.1371/journal.pgen.1007572 (PMC6101405; doi:10.1371/journal.pgen.1007572)
Supplement: S1 Table — (DOCX) [file pgen.1007572.s013.docx]

**Supplemental Information**

**Table S1. Strains used in this study**

| **Strain** | **Genotype** |
| --- | --- |
| FL58 | *h^-^ dos1Δ::ura4^+^ GFP-swi6::leu2 ura4-D18 leu1-32* |
| FL158 | *h^-^ clr4Δ::ura4^+^ cnp1-GFP::ade6^+^, ura4-D18, leu1-32, his3-D1* |
| FL510 | *h^?^ pREP1-CSE4-GFP dos1Δ::KanMX6 ade6-210 ura4-D18 his3-D1*  *leu1-32* |
| FL515 | *h^-^ pREP1-4N1C-GFP ura4-D18 ade6-210 his3-D1 leu1-32* |
| FL516 | *h^-^ pREP1-1N4C-GFP ura4-D18 ade6-210 his3-D1 leu1-32* |
| FL529 | *h^?^  pREP1-CSE4-NΔ-GFP ura4-D18 ade6-210 his3-D1 leu1-32* |
| FL554 | *h^-^  pREP1-cnp1-GFP ura4-D18 ade6-210 his3-D1 leu1-32* |
| FL571 | *h^-^  ade6^+^::cnp1-GFP ura4-D18, leu1-32, his3-D1* |
| FL593 | *h^-^ pREP1-CSE4-GFP clr4Δ::ura4^+^ ura4-D18 leu1-32* |
| FL595 | *h^-^ pREP1-CSE4-GFP mts2-1 his2 leu1-32* |
| FL634 | *h^-^ pREP-cnp1-GFP mts2-1 his2 leu1-32* |
| FL650 | *h^?^ pREP1-CSE4-GFP clr4Δ::ura4^+^ mts2-1 ura4-D18 leu1-32 his2?* |
| FL651 | *h^?^ pREP1-cnp1-GFP clr4Δ::ura4^+^ mts2-1 ura4-D18 leu1-32 his2?* |
| FL652 | *h^-^ pREP1-cnp1-GFP clr4Δ::ura4^+^ ura4-D18 leu1-32 ade6-210? his3-D1?* |
| FL801 | *h^?^ pREP1-CSE4-GFP sad1-CFP-KanMX6 ade6-M210 ura4-D18 leu1-32*  *his3-D1?* |
| FL802 | *h^?^ pREP1-CSE4-GFP cnp1-1 leu1-32 ade6-210? his3-D1?* |
| FL803 | *h^?^ empty-pREP1 cnp1-1 leu1-32 ade6-210? his3-D1?* |
| FL804 | *h^-^  pREP1-CSE4-GFP ura4-D18 ade6-210 his3-D1 leu1-32* |
| FL805 | *h^?^ scm3-19-HA-KanMX6 ade6^+^::cnp1-GFP ura4-D18 ade6-210 leu1-32*  *his3-D1* |
| FL806 | *h^?^ scm3-19-HA-KanMX6 clr4Δ:ura4^+^ ade6^+^::cnp1-GFP ura4-D18*  *leu1-32 his3-D1* |
| FL807 | *h^?^ pREP1-CSE4-GFP mCherry-swi6::ura4^+^ ura4-D18 leu1-32 ade5D*  *arg3D his5D* |
