# Supplementary material for: Heterochromatin and RNAi regulate centromeres by protecting CENP-A from ubiquitin-mediated degradation
Source: PLoS Genet. 2018 Aug 8;14(8):e1007572. doi: 10.1371/journal.pgen.1007572 (PMC6101405; doi:10.1371/journal.pgen.1007572)
Supplement: S2 Table — (DOCX) [file pgen.1007572.s014.docx]

**Supplemental Information**

**Table S2. Primers used in this study.**

| Primer name | Sequence (5'-3') |
| --- | --- |
| Act_1 | ATGGAAGAAGAAATCGCAGCG |
| Act_2 | GATGCCAAATCTTTTCCATATC |
| cnt_f | GCTAGAATAGAACAGATACCCA |
| cnt_r | ATTACAACGACGAAAGCCTC |
| GFP-f | GGAGAGGGTGAAGGTGATGC |
| GFP-r | CATAACCTTCGGGCATGGCA |
| SubT-f | CGG CTG ACG GGT GGG GCC CAA TA |
| SubT-r | GTG TGG AAT TGA GTA TGG TGA A |
| Otr-r | GAAAACACA TCG TTG TCT TCA GAG |
| Otr-f | CGT CTT GTA GCT GCA TGT GAA |
